# Surface Neuronal‐Like Structuring of High‐Strength CFRP for Enhanced Electromagnetic Interference Shielding

**DOI:** 10.1002/advs.77333

**Published:** 2026-08-20

**Authors:** Wei Cheng, Ben Jia, Shuhan Xiang, Biao Chen, Yongjun Zhang, Zhuo Liu, Xiaopeng Wan, Heyuan Huang

**Affiliations:** ^1^ School of Aeronautics Northwestern Polytechnical University Xi'an People's Republic of China; ^2^ School of Civil aviation Northwestern Polytechnical University Xi'an People's Republic of China; ^3^ Institute of Nuclear and New Energy Technology Tsinghua University Beijing People's Republic of China; ^4^ School of Mechanical & Energy Engineering Zhejiang University of Science & Technology Hangzhou People's Republic of China; ^5^ School of Mechanical Engineering Northwestern Polytechnical University Xi'an People's Republic of China; ^6^ National Key Laboratory of Aircraft Configuration Design Xi'an People's Republic of China

**Keywords:** CFRP, ductility, EMI shielding, neuronal‐like structure, synergistic promotion

## Abstract

In the functional structures of aerospace carbon fiber‐reinforced polymer (CFRP) composites, achieving the simultaneous enhancement of electromagnetic interference (EMI) shielding performance and mechanical properties remains a critical challenge. In this work, an efficient CFRP interfacial modification strategy combining electrodeposition with vacuum heat treatment is proposed to address the inherent trade‐off between shielding effectiveness and mechanical performance of CFRP‐EMI shielding materials. A neuronal‐like micro‐nano Ni coating is fabricated on carbon fiber surfaces to enhance electromagnetic shielding capacity. The formed discontinuous Ni─C miscible regions, Ni/NiO/C heterogeneous multiphases, and nanoporous structures effectively improve the interfacial integration between the matrix and coating, as well as the mechanical properties. In the X‐band, the reflection loss, absorption loss, and total EMI shielding effectiveness are increased by 33%, 18.6%, and 22.5%, respectively, while the ductility is improved by 201.78%, realizing the synergistic promotion of electromagnetic shielding and mechanical performance. Combined with experimental characterization and molecular dynamics analysis, the growth mechanism of polycrystalline phases during interfacial evolution is further clarified. This study innovatively proposes a high‐efficiency and controllable design strategy for advanced CFRP‐EMI functional materials, which provides a promising solution for the application of lightweight, high‐strength, and self‐adaptive electromagnetic shielding in the design of next‐generation multifunctional aerospace structures.

## Graphical abstract

This study elucidates the microstructural evolution at the material interface of Ni‐plated plain‐weave carbon fiber fabric following vacuum heat treatment at 500°C–900°C under different current densities, as well as the mechanism underlying the synergistic enhancement of electromagnetic shielding and ductility.

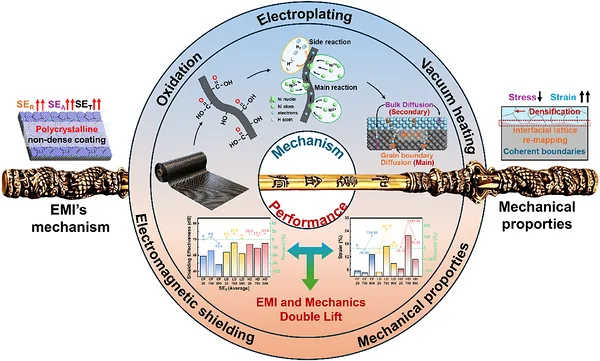

## 1 Introduction

Carbon fibers (CF) and carbon fiber‐reinforced polymer composites (CFRPs) have been widely utilized as critical materials in aerospace, national defense equipment, intelligent wearable electronics, and other fields, owing to their low density, high specific strength and specific modulus, excellent corrosion resistance, and structural designability [1, 2, 3]. Nevertheless, the rapid advancement of 5G communication and Internet of Things technologies has exacerbated EMI issues, and electromagnetic pollution has evolved into a severe threat to the stable operation of precision equipment [4, 5, 6, 7, 8]. Therefore, in response to the dual requirements of structural load‐bearing capacity and electromagnetic compatibility under extreme service conditions, the development of high‐strength CFRPs with superior EMI shielding performance has become an urgent engineering demand [9, 10].

In terms of mechanical reinforcement, mainstream strategies adopt physical modification methods such as plasma treatment to introduce polar groups and increase surface roughness, thereby strengthening mechanical interlocking between fibers and matrix [11, 12, 13, 14, 15]. Microwave irradiation etching is applied to achieve rapid surface activation, while the deposition of nanomaterials (e.g., graphene oxide, carbon nanotubes, metal–organic frameworks) establishes chemical‐physical bridging effects via their large specific surface area and abundant active groups [16, 17, 18, 19]. For the improvement of EMI shielding performance, metallized coatings are commonly introduced to construct conductive and magnetic loss pathways. Combined with elaborate structural design, such coatings enhance absorption loss and achieve high shielding effectiveness [20, 21, 22, 23, 24]. Diversified multi‐level heterogeneous structures, including hollow core‐shell Ni/C/SiO_2_ composites [25], sintered networks of porous Ni@CF paper [26], multi‐layer structures with gradient electromagnetic parameters [27], and FeSiAl integrated periodic frequency selective surfaces [28], have been constructed to realize broadband and strong electromagnetic absorption through impedance matching optimization and multiple scattering effects. However, the enhancement of mechanical properties relies on robust interfacial bonding derived from chemical bonding between fibers and matrix. In contrast, most aforementioned functionalization strategies for EMI shielding, especially the fabrication of metal coatings and heterogeneous structures, are dominated by physical assembly and mechanical interlocking. Stable chemical interfaces can hardly form on the inert CF surface, inevitably resulting in stress concentration and coating debonding. Consequently, the chemical incompatibility and structural contradiction between functional phases and load‐bearing interfaces become the core bottleneck restricting the synergistic improvement of mechanical and shielding performances.

To address the challenge of reconciling functional phases and load‐bearing interfaces, current research predominantly adopts CF surface modification strategies, primarily including nanocoating modification. For instance, the construction of composite nanocoatings such as MXene/carbon nanotube and MXene/graphene oxide [29, 30, 31, 32] and the assembly of metal–organic frameworks (MOFs) with multi‐walled carbon nanotubes (MWCNTs) [33] have achieved an EMI shielding effectiveness improvement of 10–15 dB and an interfacial shear strength enhancement of over 15% by improving stress transfer and constructing complex surface structures. However, nano coatings are prone to agglomeration, which makes achieving uniform dispersion at high loadings a challenge [34]. In addition, electroless deposition is a more widely applied synergistic reinforcement approach. Examples include electroless nickel (Ni) deposition combined with polydopamine surface modification [35] and Ni─Cu co‐deposition surface modification [36], which maintain the tensile strength of CFRP composites and improve EMI shielding effectiveness by 10–20 dB through enhancing electrical conductivity and mechanical interlocking. Nevertheless, this method suffers from tedious procedures and poor process controllability. Moreover, the mechanical interlocking between the carbon matrix and the coating still cannot fully exploit the inherent advantages of metallic coatings in terms of electrical conductivity and ductility, leaving considerable room for improvement. Notably, our previous studies have demonstrated that under appropriate temperature conditions, the interface between carbon materials and coatings can be activated, leading to the formation of coherent solid solution zones, achieving interfacial chemical bonding, and thus enhancing interfacial adhesion [37, 38]. However, direct high‐temperature sintering modification tends to cause oxidative etching and embrittlement of the carbon material skeleton [39].

Compared with electroless deposition, electrodeposition offers advantages including low cost, simple operation, precise control over coating morphology, grain size, and density via current density, and the potential for large‐scale continuous production [40]. Coupling electrodeposition with high‐vacuum linear heating (LH) heat treatment can not only fully protect the graphite skeleton of CFs from oxidative damage in a strictly oxygen‐free environment but also provide sufficient thermodynamic driving force for bidirectional interdiffusion of interfacial atoms and lattice reconstruction, inducing the formation of a chemical metallurgical bonding interface with strong chemical bonding. This approach holds potential for solving the persistent problems of easy debonding and delamination between metallic coatings and CFs.

Based on this, this work proposes an efficient interface modification strategy combining electrodeposition and high‐vacuum heat treatment. By constructing neuronal‐like micro‐nano Ni coatings on the surface of CFs, the EMI shielding effectiveness is improved. Meanwhile, the formed discontinuous Ni─C interdiffusion zones, Ni/NiO/C heterogeneous multiphase interfaces, and nanoporous structures enhance the integrity of the matrix‐coating system and significantly improve the mechanical properties, ultimately achieving a synergistic breakthrough in both EMI shielding and mechanical performance. Combined with multi‐scale characterizations including X‐ray diffraction (XRD), Raman spectroscopy, and X‐ray photoelectron spectroscopy (XPS), Focused ion beam (FIB), Transmission electron microscope (TEM), as well as molecular dynamics simulations, the mechanisms of interfacial atomic diffusion and coating growth are further elucidated. This work demonstrates an efficient and tunable design method for EMI shielding CFRP materials and provides a promising pathway for the development of next‐generation lightweight, high‐strength, and multifunctional CF structures for aerospace applications.

## 2 Results and Discussions

### 2.1 Design Inspiration and Control of Electroplating Morphology

**FIGURE 1:**
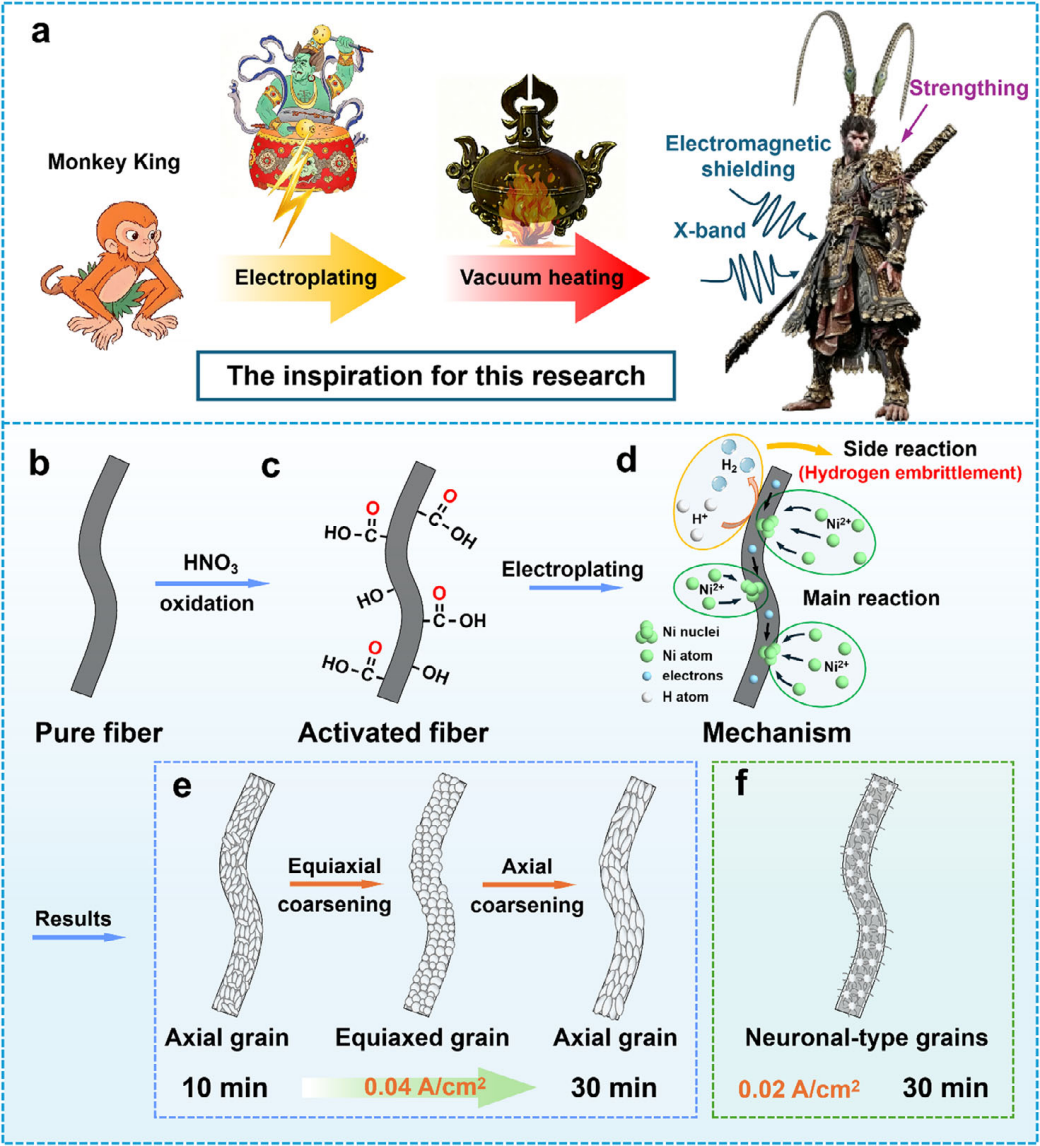
(a) Schematic diagram of the design inspiration derived from the growth experience of the mythological figure Sun Wukong; (b) pristine CF; (c) schematic illustration of the CF surface activated by concentrated nitric acid; (d) schematic diagram of Ni electroplating onto the CF; (e) time‐dependent growth model of the Ni coating at a current density of 0.04 A/cm^2^; (f) growth model of the Ni coating after electrodeposition for 30 min at a current density of 0.02 A/cm^2^.

The design concept, surface activation mechanism, and morphological evolution of the Ni coating are intuitively and systematically presented in Figure 1. Figure 1a illustrates the cultural metaphor and design inspiration of this study: inspired by the growth narrative of Sun Wukong—the Monkey King in traditional Chinese mythology—who, after wreaking havoc in the Heavenly Palace, endured thunder and lightning strikes and was tempered in the Eight Trigrams Furnace, ultimately attaining a body as hard as copper and iron and fiery golden eyes. In this work, “thunder and lightning strikes” metaphorically represent the electroplating process, while “tempering in the Eight Trigrams Furnace” symbolizes the vacuum high‐temperature heat treatment, thereby achieving the synergistic enhancement of both EMI shielding and plastic toughness of the composite.

Figure 1b,c elucidate the activation mechanism of the CF before metallization. The pristine CF surface is chemically inert, and direct electroplating readily leads to nucleus agglomeration and coating delamination. Through liquid‐phase etching with concentrated HNO_3_, abundant polar oxygen‐containing functional groups, such as ─OH and ─COOH, are introduced onto the carbon surface, serving as adsorption anchoring sites for Ni electroplating. These groups guide the uniform attachment and nucleation of Ni^2^^+^ ions on the fiber surface, as displayed in Figure 1d. Meanwhile, the hydrogen evolution side reaction inherent in the electroplating process poses a risk of hydrogen embrittlement to the CF substrate.

**FIGURE 2:**
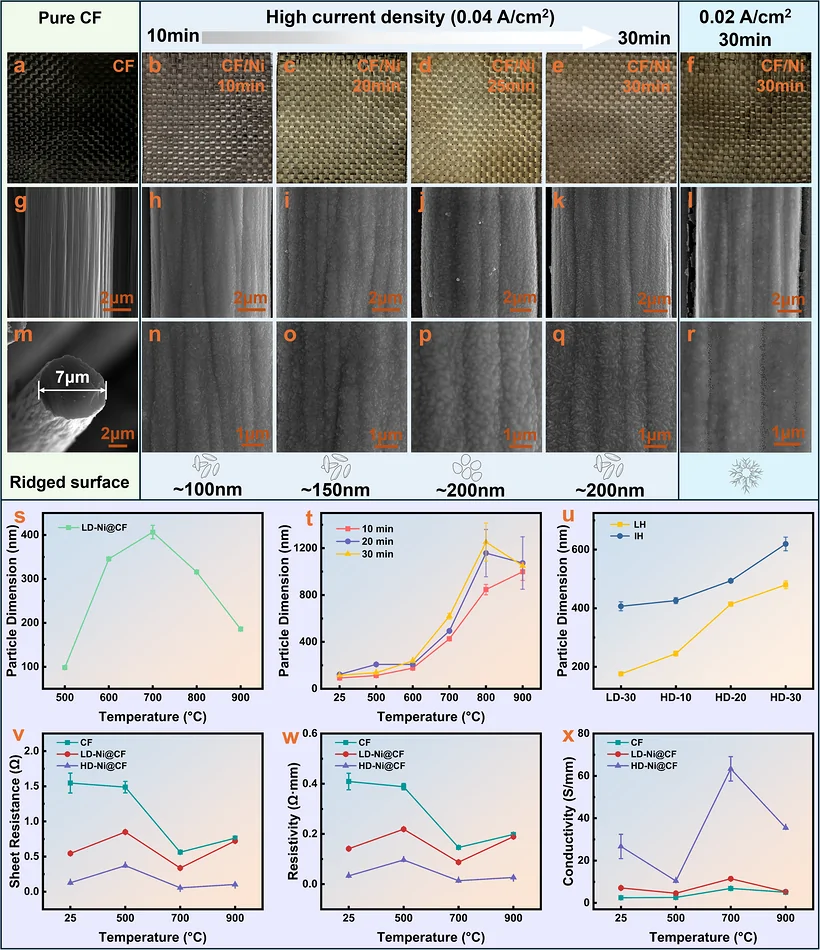
Macroscopic morphology and SEM images of the pristine CF plain weave fabric (a, g, m), and after electrodeposition at 0.04 A/cm^2^ for 10 min (b, h, n), 20 min (c, i, o), 25 min (d, j, p), and 30 min (e, k, q), together with the macroscopic morphology and SEM images after electrodeposition at 0.02 A/cm^2^ for 30 min (f, l, r); Variation of coating grain size with heat treatment temperature for the low current density (LD) sample (s); Variation of grain size with heat treatment temperature for samples prepared with different electrodeposition durations under high current density (HD) (t); Comparison of the effects of LH and instantaneous heating (IH) processes on the grain size of different coatings at 700°C (u); Variation curves of sheet resistance, resistivity and electrical conductivity with heat treatment temperature for pristine CFs, LD‐coated and HD‐coated samples (v–x).

The initial quality of the electroplated coating is a prerequisite for determining the success of the subsequent strong interface bonding. By integrating the macro‐ and micro‐morphologies presented in Figures 1e,f and  2 with the grain size analysis in Figure S1, thickness measurements in Figure S2, and energy‐dispersive spectroscopy (EDS) results in Figure S3, this study systematically elucidates the regulatory effects of processing parameters on coating growth. The pristine T300 CF surface exhibits distinct axial groove structures, with a diameter of approximately 7 µm. Under an HD of 0.04 A/cm^2^, the ample electrochemical driving force provided by the elevated current renders the crystal nucleation rate greater than the growth rate. After electroplating for 10 min, Ni crystal nuclei approximately 91 nm in size uniformly precipitate on the fiber surface with an axial distribution (coating thickness ∼109 nm, Figure S2f); at 20 min, the grains grow to 122 nm, and the coating thickness reaches 178 nm (Figure S2g), displaying metallic luster. When the electroplating time is increased to 25 min, the grains undergo equiaxed coarsening to 175 nm with a coating thickness of 266 nm (Figure S2h), forming a highly dense, continuous, and pore‐free bright golden coating. At this stage, an optimal balance between coating continuity and compactness is attained. Further extending the time to 30 min leads to grain refinement to 115 nm and a substantial thickness increase to 428 nm (Figure S2i), with the surface exhibiting a silvery‐golden metallic luster. In contrast, under the LD of 0.02 A/cm^2^ process, ion reduction proceeds slowly, and the crystal growth rate surpasses the nucleation rate. After 30 min of deposition, the coating reaches a thickness of ∼322 nm (Figure S2j), and the Ni grains exhibit a neuronal‐like growth tendency (Figures 1f and 2r). This non‐uniform kinetics results in extremely thin coatings at the fiber grooves and even the formation of micro‐ and nano‐scale hollow pores, yielding a macroscopically mottled dark brown luster. EDS elemental mapping, as shown in Figure S3, further corroborates the local inhomogeneity of elemental distribution. In summary, HD deposition for 25–30 min constitutes the optimal process for continuous Ni coating, establishing an ideal physical contact foundation for subsequent interfacial diffusion. Nonetheless, the non‐dense, neuronal‐like structure generated by LD deposition offers favorable interfacial conditions for electromagnetic multiple reflection and absorption, thereby possessing considerable EMI shielding potential. Statistical analysis of coating grain sizes under various heat treatment conditions is presented in Figure 2s–u, and the detailed grain size distributions are provided in Figures S1 and S4–S6 of the Supporting Information. The results reveal that thermally induced Ni grain growth exhibits strong temperature‐dependent and process‐dependent behaviors.

For LD coatings, grain size increases moderately within the temperature range of 500°C–600°C and peaks at approximately 410 nm at 700°C. Upon heating to 900°C, severe spheroidization and fragmentation occur throughout the Ni coating, which destroys the structural continuity; Consequently, the average grain size declines to below 200 nm. This trend is fully consistent with the coating degradation observed via SEM morphological characterization.

For HD coatings, grain size shows an overall monotonic upward trend with prolonged electrodeposition duration and elevated heat treatment temperature. For samples deposited for 30 min, the grain size exceeds 1200 nm after 900°C treatment, which verifies the sustained thermal coarsening of dense Ni coatings at high temperatures.

Comparison between the two heating protocols indicates that LH mitigates thermal shock and provides sufficient relaxation time for homogeneous atomic diffusion. All specimens treated via LH possess smaller average grain sizes and narrower size distributions than those subjected to rapid IH, demonstrating that slow LH effectively suppresses abnormal grain growth. Such a regulatory effect is particularly prominent for thin Ni coatings.

### 2.2 Mechanisms of Coating Restructuring and Microstructural Evolution

**FIGURE 3:**
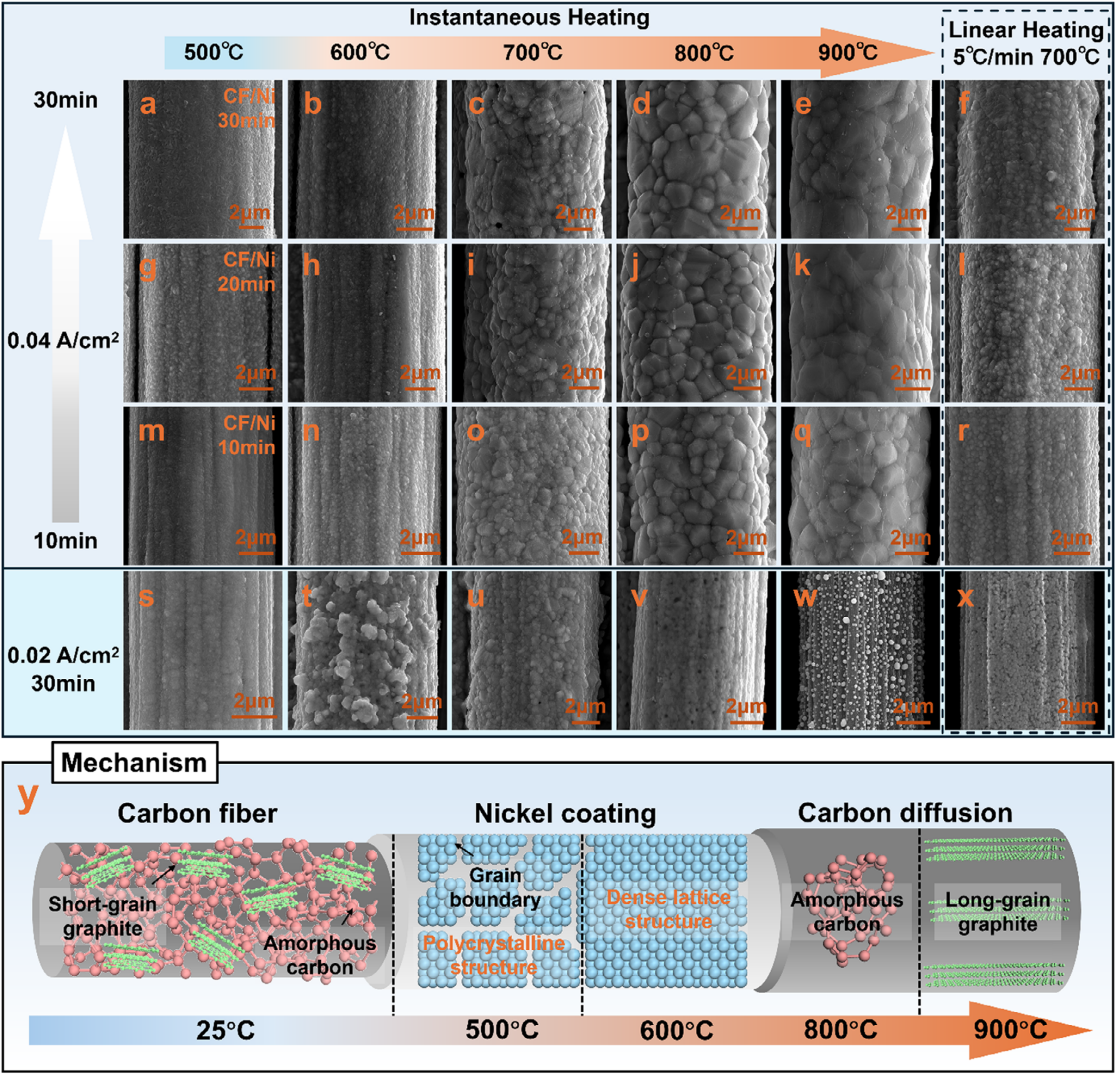
Microstructural morphologies of samples electrodeposited at 0.04 A/cm^2^ for 30 min (a–e), 20 min (g–h), and 10 min (m–q), and that at 0.02 A/cm^2^ for 30 min (s–w) after IH to 500°C–900°C followed by vacuum heat treatment for 2 h; microstructural morphologies of samples electrodeposited at 0.04 A/cm^2^ for 30 min (f), 20 min (l), and 10 min (r), and that at 0.02 A/cm^2^ for 30 min (x) after LH to 700°C followed by vacuum heat treatment for 2 h; (y) schematic model of the evolution mechanism of the heterogeneous interface with increasing temperature.

To elucidate the thermally driven evolution of the Ni─C interface, this study first employed SEM, as shown in Figure 3, combined with detailed grain size statistics presented in Figures S4–S6, to systematically evaluate the microstructural morphology and grain growth kinetics under different heat treatment conditions.

For the dense initial coating prepared by HD electroplating for 30 min, the microstructural morphology exhibited a pronounced temperature dependence. During the low‐temperature densification stage at 500°C–600°C, the diffusion activity of Ni atoms remained relatively low. The sample treated at 500°C retained an average grain size of approximately 136.3 nm, as shown in Figure S6o, essentially preserving the fine‐grained characteristics of the as‐plated state. When the temperature was increased to 600°C, the grains grew to approximately 237.2 nm (Figure S6l), accompanied by localized coalescence, while the coating surface remained continuous, intact, and free of obvious porosity. A critical morphological transition occurred at 700°C, where Ni atoms acquired sufficient diffusion of activation energy, leading to the mutual fusion of grain boundaries and the formation of a continuous, compact metallic layer. Notably, the heating mode exerted a decisive regulatory role at this stage. IH resulted in rapid grain coarsening to approximately 619.3 nm (Figure S6i) with a broad size distribution. In contrast, the LH process at a rate of 5°C/min provided sufficient relaxation time for homogeneous diffusion of Ni atoms, ultimately yielding a uniform Ni layer with an average grain size of approximately 479.7 nm (Figure S5c), a narrow size distribution, and enhanced compactness. As the temperature further soared to 800°C–900°C, excessive thermodynamic responses occurred. At 800°C, the grains coarsened drastically to approximately 1.25 µm (Figure S6f), exhibiting distinct polyhedral characteristics accompanied by a significant increase in surface roughness. At 900°C, the average grain size was approximately 1.05 µm (Figure S6c), and a suspected outwardly protruding carbon recrystallization overlayer was clearly observable on the surface.

In contrast, the coating prepared under LD, owing to the intrinsic porosity in its initial structure, displayed inferior thermal shock resistance. Quantitative image statistical analysis on the as‐deposited sample fabricated at LD reveals a surface porosity of 17.33% and an average pore size of 84.74 ± 4.35 nm. Combined with cross‐sectional TEM characterization results presented in Figure S10, pores at this scale only exist on the outermost coating surface, originating from surface undulations induced by inhomogeneous grain growth during electrodeposition. The Ni coating maintains an overall continuous and compact structure without penetrating holes, and the CF substrate is uniformly covered by the deposited Ni layer. At 500°C, the grain size was 98.1 nm, as shown in Figure S4a; however, severe grain agglomeration occurred at 600°C, with the grain size jumping to approximately 345.3 nm, as shown in Figure S4b, and a notable increase in surface roughness was observed. After IH treatment at 700°C, although the interfacial state was stabilized due to the fulfillment of thermal threshold conditions for the interfacial evolution between the coating and the CF, and a continuous, non‐dense coating structure was on the surface, visible bare CF sites remained. Notably, at LH treatment at 700°C, the surface of the CF presents a spherical dense arrangement. The adjacent spherical clusters are linked together by nanofibers. Quantitative statistical results demonstrate that the sample subjected to this treatment exhibits an elevated surface porosity of 31.26% with an increased average pore size of 232 nm ± 8.43 nm. The growth in pore proportion and dimension originates from the synergistic effects of bidirectional interfacial atomic interdiffusion, in situ reduction of NiO, and coating structural reconstruction during heat treatment. Such micro‐nano porous architecture matches well with the discontinuous Ni coating. On one hand, abundant pores are introduced inside the coating to strengthen interfacial polarization under alternating electromagnetic fields. On the other hand, incident electromagnetic waves undergo multiple reflection and scattering within pore cavities, extending the wave propagation path and energy dissipation process, which provides a critical structural foundation for the enhancement of electromagnetic absorption loss. When the temperature was elevated to the extremely high range of 800°C–900°C, the bidirectional diffusion disrupted the continuous metallic framework. At 800°C, a large number of exposed sites appeared on the coating surface; at 900°C, the Ni grains underwent spheroidization, with their size shrinking and degenerating into isolated Ni particles, exhibiting an average size of merely 185.8 nm. These particles adhered to the exposed CF surface, completely losing structural continuity and the conductive network. It is worth noting that a large number of molten pits appeared on the surface of the CF, which proved that under the effect of high temperature, the Ni plating layer had a catalytic etching effect on the surface structure of the CF.

Based on the above objectively observed multiscale morphological and grain size evolution, this study further employed the mechanistic model illustrated in Figure 3y to systematically integrate and elucidate the deep microscopic evolution mechanism of the Ni─CF interface from physical anchoring to coherent chemical interface reconstruction and ultimately to carbon recrystallization. At room temperature (RT), the pristine CF substrate was composed of intertwined short‐range ordered graphitic microcrystalline and a substantial amount of amorphous carbon (AC), while the electrodeposited Ni layer on its surface exhibited a typical polycrystalline structure with high‐density grain boundaries. During the 500°C–600°C stage, the polycrystalline Ni layer gradually reorganized into a denser lattice structure as local grain boundary healing proceeded, yet the interface still predominantly featured mechanical/physical bonding without significant deep interdiffusion. When the thermodynamic driving force surpassed the activation threshold of approximately 700°C, the Ni coating layer underwent a critical transition to chemical bonding: polycrystalline grain boundaries merged, and crystallite rapid growth occurred. The linear ramping heating mode effectively suppressed localized stress concentration and abnormal grain growth, thereby forming an optimally uniform coherent chemical network. As the temperature rose to 800°C, the system entered a high‐temperature disordered carbon diffusion stage, wherein a large quantity of activated AC atoms within the CF commenced outward spillover toward the Ni layer. At the extremely high temperature of 900°C, under the catalytic effect of the dense Ni layer, these AC atoms that had diffused to the coating surface underwent lattice rearrangement and ultimately recrystallized into a highly ordered long‐range graphitic phase. This mechanistic model not only coincides with the recrystallization characterization phenomena observed on the surface of HD samples at 900°C, but also fundamentally reveals the origin of coating spheroidization in LD samples: their non‐dense initial grain boundaries deprive the high‐temperature carbon diffusion of effective physical confinement and a uniform catalytic substrate, eventually leading to the spheroidization of the metallic Ni framework.

### 2.3 Mechanisms of Lattice Structure Evolution

**FIGURE 4:**
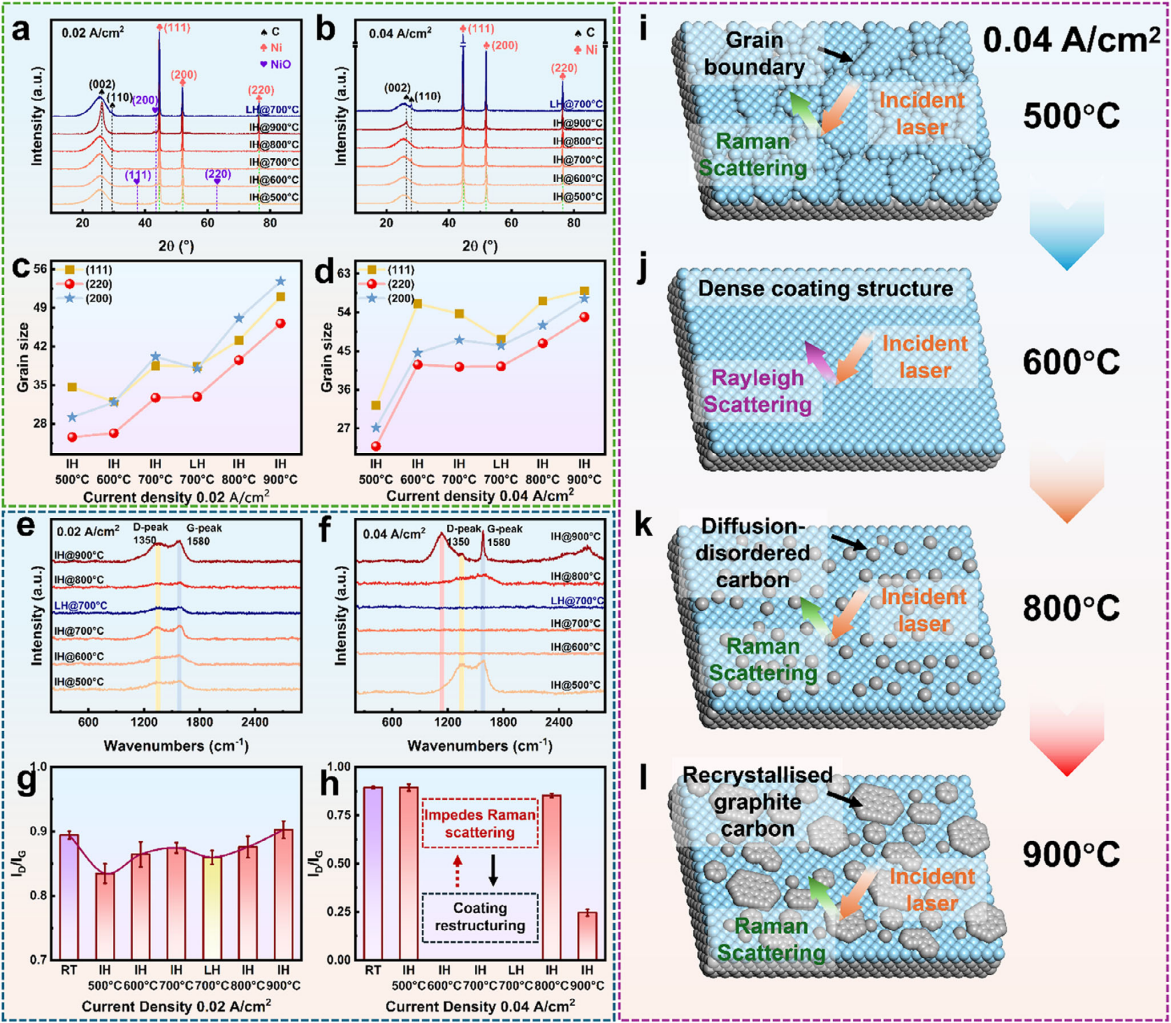
XRD patterns and Ni grain growth kinetic curves calculated using the Scherrer equation for Ni‐coated CF plain weave fabric prepared at current densities of 0.02 A/cm^2^ (a, c) and 0.04 A/cm^2^ (b, d); Raman spectra and I_D_/I_G_ intensity ratio statistics for Ni‐coated CF plain weave fabric at 0.02 A/cm^2^ (e, g) and 0.04 A/cm^2^ (f, h); schematic illustration of the structural evolution mechanism of the coating on the CF surface at a current density of 0.04 A/cm^2^ (i–l).

XRD and Raman spectroscopy were further employed to conduct an in‐depth analysis of the dynamic structural evolution of both the Ni coating and the CF surface during heat treatment, as presented in Figure 4.

As revealed by Figure 4a, during the LD coating process, in the 500°C–600°C range, the XRD patterns exhibited weak NiO characteristic peaks [41] at 37.2°, 43.3°, and 62° in addition to the typical Ni and C diffraction peaks, primarily originating from the oxidation reaction of trace oxygen adsorbed on the electrodeposited Ni surface during the initial stage of vacuum heating. While not appearing in the HD coating process as shown in Figure 4b, due to the strong signal of the Ni characteristic peak, the signal of the NiO peak was masked. A critical crystallographic transition occurred at 700°C: the NiO characteristic peaks completely vanished, providing evidence that diffusing C atoms at the interface triggered an in situ redox reaction at elevated temperature, reducing NiO to metallic Ni. This reaction liberated many active Ni atoms, thereby promoting the diffusion of Ni into the carbon substrate. Concurrently, the C(002) diffraction peak strengthened and sharpened with increased temperature, both in LD and HD coating processing. In addition, the C(110) characteristic peak became prominently visible above 700°C, directly reflecting that carbon atoms, catalytically activated by the Ni coating, detach from the CF skeleton and undergo structural reorganization. Especially in the LH treatment processing, which exhibited superior interfacial catalytic activity. When the temperature was raised to 900°C, the C(002) peak underwent drastic sharpening, confirming that this temperature, under the combined effect of high temperature and strong catalysis by metallic Ni, constitutes the critical point for the rearrangement of the disordered CF structure and long‐range graphitization recrystallization, consistent with the SEM observations reported in the preceding section.

To quantitatively elucidate the grain size evolution of Ni, the Scherrer equation [42] was applied to the principal diffraction peaks of Ni, namely (111), (200), and (220):

(1) $$D=\frac{K\lambda }{\beta COS\theta }$$

In the Equation 1, where D is the average crystallite size, K is the Scherrer constant (taken as 0.89 in this work), λ is the incident X‐ray wavelength (Cu Kα radiation, λ = 0.154056 nm), β is the full width at half maximum (FWHM) of the diffraction peak after correction for instrumental broadening (converted to radians), and θ is the Bragg diffraction angle of the corresponding crystal plane. The calculated results, shown in Figure 4c,d, revealed distinct kinetic differences in grain growth depending on the electrodeposition conditions. For the LD sample, the grains predominantly grew along the normal direction of the (200) plane; the size changed only slightly below 600°C and displayed a monotonically increasing trend above 700°C. In contrast, the dense precursor coating fabricated at HD exhibited stronger XRD signals and a preferred orientation along the (111) plane. Its grains commenced rapid growth as early as 600°C, yet the size increase entered a plateau at 700°C, followed by a near‐linear growth from 800°C to 900°C. It is worth emphasizing that, compared with IH, the LH mode provided the system with sufficient stress relaxation time, thus significantly suppressing the abnormal grain coarsening induced by high temperature. Under this regime, the Ni diffraction peaks were relatively weaker, and the grains remained fine and uniform, whereas the C(110) peak, which signifies interfacial interdiffusion, was more intense. This indicates that the fine‐grained network not only preserves the flexible buffering and coordination capabilities of the coating but also provides a high density of grain boundaries to promote large‐area in situ Ni─C diffusion.

Raman spectroscopy further characterized the degree of carbon surface disorder and the lattice repair mechanism, as displayed in Figure 4e–h. The D band located near 1350 cm^−^^1^ corresponds to disordered carbon (sp^3^ defect hybridization), while the G band near 1580 cm^−^^1^ corresponds to graphitized carbon (sp^2^ hybridization) [43]; their intensity ratio I_D_/I_G_ was adopted to quantitatively evaluate the degree of disorder in the carbon structure. The pristine RT sample, having undergone intense oxidative etching in concentrated nitric acid, featured a high density of oxygen‐containing functional groups on its surface, and consequently exhibited a high I_D_/I_G_ value. In the LD group, as shown in Figure 4e,g, the coating was discontinuous, locally exposing the substrate; at 500°C, Ni nucleation preferentially occupied high‐energy defect sites, leaving the carbon lattice locally more intact and yielding a recorded minimum in I_D_/I_G_. By comparison, the HD group demonstrated a unique interfacial shielding effect, as shown in Figure 4f,h. At 500°C, the degree of carbon disorder remained close to the room‐temperature level, as shown in Figure 4i, with the surface Ni coating exhibiting a non‐dense structure. During the transitional heating stage at 600°C–700°C, the compact and thick uniform Ni layer almost completely shielded the Raman scattering signals of the underlying carbon, causing both the D and G bands to disappear, as presented in Figure 4j. This phenomenon not only confirms the high compactness of the coating but also indicates that the microscopic diffusion of carbon atoms was strictly confined within the interfacial solid‐solution region. Only when the heat temperature exceeded the extreme threshold of 800°C did the characteristic Raman bands of carbon reappear, marking the activation of bulk out‐diffusion, as shown in Figure 4k. Upon reaching 900°C, the I_D_/I_G_ value declined significantly, substantiating that the high‐energy disordered carbon atoms that had diffused outward overcame the activation energy barrier under the strong catalytic effect of the high‐temperature metallic Ni and underwent large‐scale graphitization recrystallization, as illustrated in Figure 4l. This conclusion forms a closed‐loop corroboration with the previously observed sharpening of the C(002) diffraction peak in XRD at 900°C.

**FIGURE 5:**
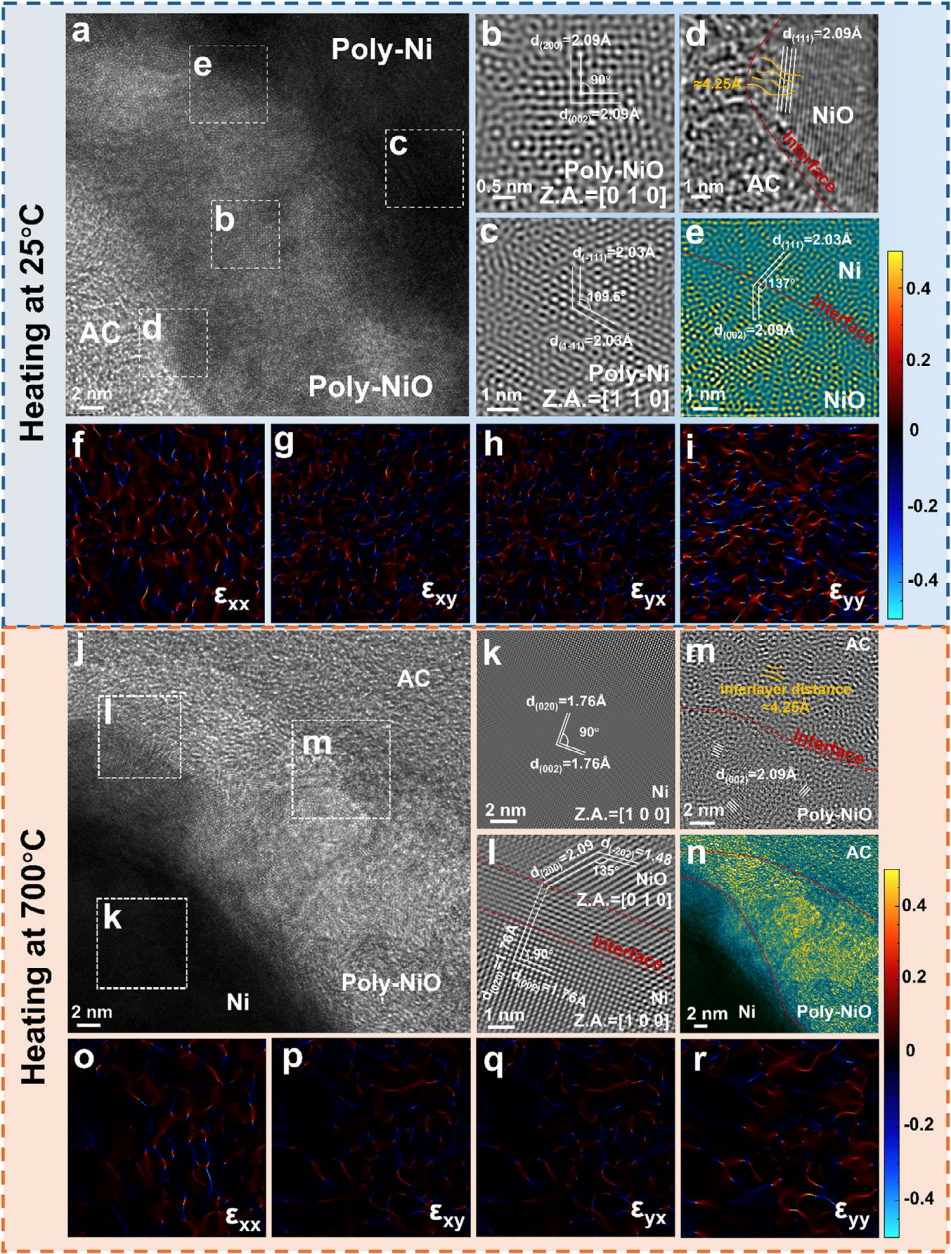
High‐resolution TEM (HRTEM) characterizations and geometric phase analysis (GPA) of strain fields at the Ni/NiO/AC triple‐phase interfaces of LD as‐deposited samples and specimens subjected to LH treatment at 700°C. (a–e) High‐resolution bright‐field TEM images of triple‐phase interfaces of room‐temperature as‐deposited samples and the corresponding inverse fast Fourier transform (IFFT) lattice images of selected regions; (f–i) Distributions of strain tensors (ε_xx_, ε_xy_, ε_yx_, ε_yy_) in the interfacial regions of room‐temperature as‐deposited samples; (j–n) High‐resolution bright‐field TEM images of triple‐phase interfaces of samples after LH treatment at 700°C and the corresponding IFFT lattice images of selected regions; (o–r) Distributions of strain tensors in the interfacial regions of samples after 700°C heat treatment.

**FIGURE 6:**
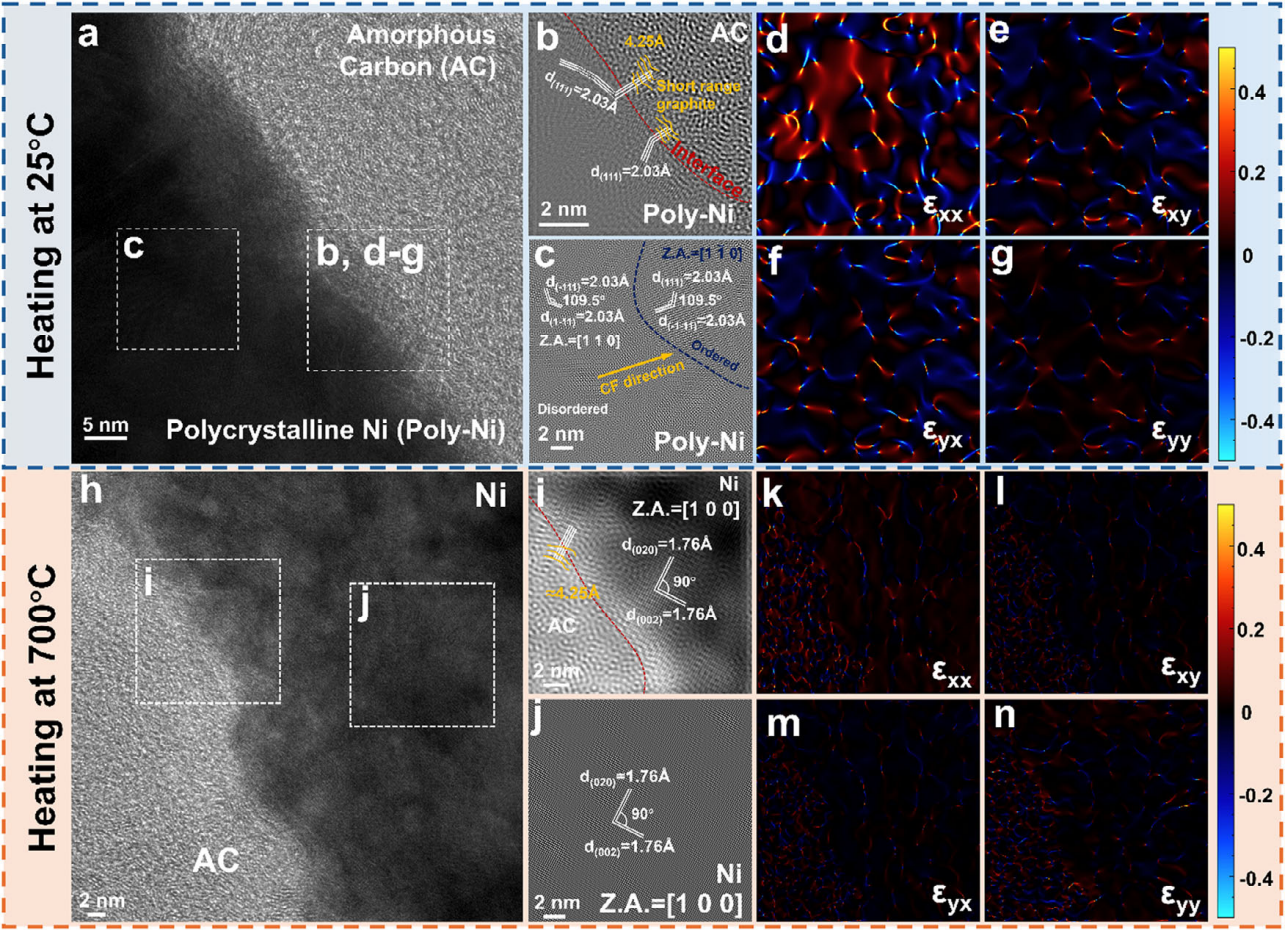
HRTEM characterization and GPA of strain at the Ni/AC two‐phase interface in the LD as‐plated sample and the sample LH treated to 700°C: (a–c) High‐resolution bright‐field images of the two‐phase interface in the RT as‐plated sample, corresponding selected‐area IFFT lattice images, and (d–g) interfacial strain tensor maps of (a); (h–j) High‐resolution bright‐field images of the two‐phase interface in the sample LH treated to 700°C, corresponding selected‐area IFFT lattice images, and (k–n) interfacial strain tensor maps of (h).

To elucidate the lattice reconstruction and phase evolution behaviors of the Ni/C interface during heat treatment at the atomic real‐space scale, and to bridge the spatial resolution limitations of statistical characterizations such as XRD and Raman spectroscopy, HRTEM was employed. The typical interfaces of the LD as‐plated sample and the sample linearly heat‐treated to 700°C were characterized. Combined with GPA to quantitatively resolve the evolution of the interfacial lattice strain fields, a comparative analysis was conducted specifically on the Ni/NiO/AC three‐phase interface and the Ni/AC two‐phase interface. The results are shown in Figures 5 and 6.

As shown in Figure 5a–e, the surface oxygen‐rich defect region of the as‐plated sample exhibits a typical three‐layer structure, which, from the surface to the interior, consists of an electrodeposited polycrystalline Ni layer, a polycrystalline NiO transition layer, and the AC matrix of the CF. The interfaces between these phases are clear and sharp, with a certain degree of atomic interdiffusion observed, indicating that the as‐plated interface is dominated by physical contact and mechanical interlocking. IFFT lattice analysis of the selected areas of each phase reveals that the polycrystalline Ni region possesses a face‐centered cubic structure, displaying lattice fringes of the (1$\bar{1}$1) and ($\bar{1}$11) planes along the [110] zone axis, in Figure 5c, with an interplanar spacing of ∼0.203 nm. The grain orientations are randomly distributed with a high grain boundary density. The polycrystalline NiO layer exhibits (200) and (002) lattice fringes along the [010] zone axis, in Figure 5b, with an interplanar spacing of ∼0.209 nm. The orientation relationship can be indexed as (002)_NiO_//(111)_Ni_ with a lattice mismatch of lattice parameters of 2.87%, which indicates the formation of a coherent interface between the Ni‐rich layer and NiO‐rich layer. In the interfacial region between NiO and AC, in Figure 5d, a small amount of short‐range ordered graphite‐like crystallites (indicated by yellow lines) is present in the AC matrix. Their layer direction is approximately perpendicular to the (111) plane direction of NiO (indicated by white lines). The two phases undergo preliminary lattice interweaving at the interface, providing a structural precursor for the evolution of the interlocked structure during subsequent heat treatment. The AC region lacks long‐range ordered lattice features and contains only short‐range ordered carbon clusters, which is consistent with the intrinsic structure of pristine CFs.

The GPA results are shown in Figure 5f–i. The strain field at the as‐plated three‐phase interface exhibits localized characteristics. The amplitudes of the normal strains ε_xx_ and ε_xy_ can reach over 0.4, while the shear strains ε_yx_ and ε_yy_ are distributed in a disordered manner. The high‐strain regions are primarily concentrated within the internal regions of Ni, NiO, and the AC phase. This intra‐phase stress primarily arises from the growth behavior of polycrystalline Ni and NiO during electrodeposition. When the coating develops into a dense and continuous structure, lattice mismatch and growth constraints between adjacent grains induce pronounced stress concentration at grain boundaries.

Following vacuum heat treatment with LH to 700°C, the three‐phase interface structure undergoes significant evolution, with the corresponding high‐resolution morphology and lattice analysis shown in Figure 5j–n. The initially continuously distributed NiO transition layer is reduced in situ by active C atoms at the interface at high temperatures, undergoing fragmentation and partial consumption. It transforms into nanoscale dispersed phases distributed within the interdiffusion region of Ni and C, ultimately forming a multiphase coexisting Ni/NiO/C heterogeneous interface structure. Simultaneously, the Ni grains undergo thermally induced coarsening, leading to a decreased grain boundary density and an enhanced degree of lattice ordering. The change in grain size is consistent with the calculation results derived from the XRD Scherrer equation. Ni atoms diffuse toward the carbon matrix side along the interlayer gaps and defect channels of the CFs, while carbon atoms also undergo limited migration toward the coating side, resulting in a continuous transition characteristic of the interfacial lattice.

In the near‐interface region of the carbon matrix, the AC undergoes structural rearrangement under the catalytic action of metallic Ni, forming more short‐range ordered graphite‐like microcrystalline domains with an interlayer spacing of approximately 0.425 nm. As shown in Figure 5l, at the Ni/NiO interface, the transformation of NiO into metallic Ni via lattice relaxation is directly observed. Meanwhile, as shown in Figure 5m, the polycrystalline NiO structure exhibits pronounced grain refinement at the NiO/AC interface. This phenomenon demonstrates that the NiO coating undergoes a reduction reaction with carbon atoms to form metallic Ni, which subsequently diffuses outward toward the coating exterior and grows into a Ni layer through relaxation‐mediated growth. Notably, an increase in short‐range ordered graphite‐like structures is observed within the CF matrix in Figure 5m. This phenomenon arises from the structural catalytic effect of the transition‐metal Ni coating on the AC structure, which is in good agreement with the conclusion of enhanced graphitization degree derived from Raman spectroscopy measurements.

The corresponding quantitative results of the interfacial strain fields are shown in Figure 5o–r. After heat treatment, the overall interfacial strain level decreases significantly, the proportion of localized high‐strain concentration areas is reduced, and the strain distribution tends to become uniform. The peak value of the normal strain drops to approximately 0.2, and the degree of disorder in the shear strain is attenuated. This result confirms that interfacial atomic interdiffusion and lattice reconstruction effectively relax the as‐plated residual stress, providing a structural foundation for the enhancement of the mechanical toughness of the composite. Furthermore, the dispersedly distributed NiO nanophases form many dielectric heterojunctions with the Ni matrix and the carbon matrix. Under alternating electromagnetic fields, these can induce interfacial polarization and space charge polarization effects, acting as an important microstructural origin for the enhancement of electromagnetic wave absorption loss.

For the Ni/AC two‐phase interface on the flat regions of the CF surface, the evolution behavior differs from that of the three‐phase interface. The relevant characterization results are presented in Figure 6.

As shown in Figure 6a–c, the Ni side in the as‐plated state features a fine electrodeposited polycrystalline structure predominantly oriented along the (111) plane with an interplanar spacing of ∼0.203 nm, exhibiting a high density of grain boundaries and lattice defects. Meanwhile, the side closer to the AC structure has a more complete crystal lattice structure, which reflects the growth process of the Ni coating from the inside out. The two‐phase interface is straight and distinct, with a few transition layers present. At the interfacial boundary, shown in Figure 6b, the short‐range carbon clusters in the AC and the (111) planes of Ni exhibit a certain orientational discrepancy. However, due to the low degree of structural ordering in the carbon, no obvious interlocked features are formed. The GPA results, presented in Figure 6d–g, reveal a distinct discontinuity in the strain distribution at the interface. The normal strain undergoes an abrupt transition at the phase boundary, and the high‐strain region is linearly distributed along the interface. This reflects the interfacial stress mismatch induced by the inherent physical property differences between the metal and the carbon. Under external loading, such stress concentration sites can easily act as initiation and propagation points for cracks, constituting a significant factor in the brittle fracture characteristics of the as‐plated composites.

The microstructure of the two‐phase interface after heat treatment at 700°C is displayed in Figure 6h–j. The Ni grains undergo significant growth and grain boundary migration. Along the [100] zone axis, (020) and (002) lattice fringes are visible with an interplanar spacing of ∼0.176 nm, indicating a reduced lattice defect density and improved crystalline integrity. Interfacial atomic interdiffusion blurs the originally relatively straight phase boundary, forming an interdiffusion transition layer approximately 2–3 nm in width, characterized by a continuous and gradual lattice transition. As depicted in Figure 6i, the layers of the graphite‐like crystallites, formed via rearrangement near the interface, exhibit a perpendicular orientation relationship with the (020) planes of metallic Ni. These carbon microcrystals embed into the interfacial defects of the metal lattice, forming a tightly interlocked interface. This configuration shifts the interfacial load transfer mechanism from pure interfacial friction to the synergistic effect of mechanical interlocking and interface bonding. This effectively dissipates stress concentrations during the fracture process.

The corresponding strain field distribution is presented in Figure 6k–n. Following the heat treatment, the abrupt strain transition at the interface disappears, yielding a continuous and smooth transition. The overall strain level is significantly reduced, indicating effective relaxation of the interfacial residual stress. Such structural evolution enhances the efficiency of interfacial load transfer, dissipating fracture energy through interfacial slip and grain plastic deformation under external forces. This subsequently improves the fracture toughness of the composites, aligning with the experimental observations of enhanced plasticity in macroscopic tensile tests.

In summary, the HRTEM and GPA characterization results from Figures 5 and  6 unveil the thermally induced evolution mechanism of the Ni/C interface at the atomic scale: The as‐plated interface consists of a coherent chemical interface of Ni/NiO and a small amount of mechanical interlocking between metal and AC, exhibiting substantial stress concentration. Through interfacial atomic interdiffusion, partial reduction of NiO, and catalytic ordering of the carbon structure, the LH treatment to 700°C constructs a lattice‐interlocked structure where graphitic carbon and metallic phases are vertically embedded. This forms a blend interface with gradient transition characteristics, alongside the localized formation of a Ni/NiO/C heterogeneous multiphase structure. This structural evolution synergistically enhances interfacial bonding strength and mechanical toughness via stress relaxation and mechanical interlocking. Concurrently, it augments electromagnetic wave absorption loss through heterogeneous interfacial polarization, providing an atomic‐level experimental basis for the simultaneous enhancement of electromagnetic shielding and mechanical properties.

### 2.4 Mechanisms of Interfacial Chemical State Evolution

Nanoscale characterizations on the initial interfacial microstructure and elemental distribution of the as‐deposited LD specimens were carried out via transmission electron microscopy coupled with energy‐dispersive X‐ray spectroscopy (TEM‐EDS), as displayed in Figures S10 and S11. The results reveal a distinct phase boundary between the Ni coating and CF substrate in the as‐deposited state, accompanied by a remarkably heterogeneous distribution of oxygen at the interface. Specifically, oxygen is heavily accumulated at axial grooves and defective sites on CF surfaces, forming oxygen‐rich transition layers with a thickness of tens of nanometers; in contrast, sparsely distributed low‐concentration oxygen is observed on relatively flat fiber surface regions. This elemental distribution pattern matches the mechanism of preferential oxidative etching at defective sites during concentrated nitric acid activation. The abundant oxygen‐containing functional groups and oxidation products concentrated at the interface serve as initial reaction sites and material precursors for interfacial redox reactions, atomic interdiffusion, and chemical bond reconstruction during subsequent heat treatment and directly lead to regional discrepancies in interfacial structural evolution upon thermal processing.

**FIGURE 7:**
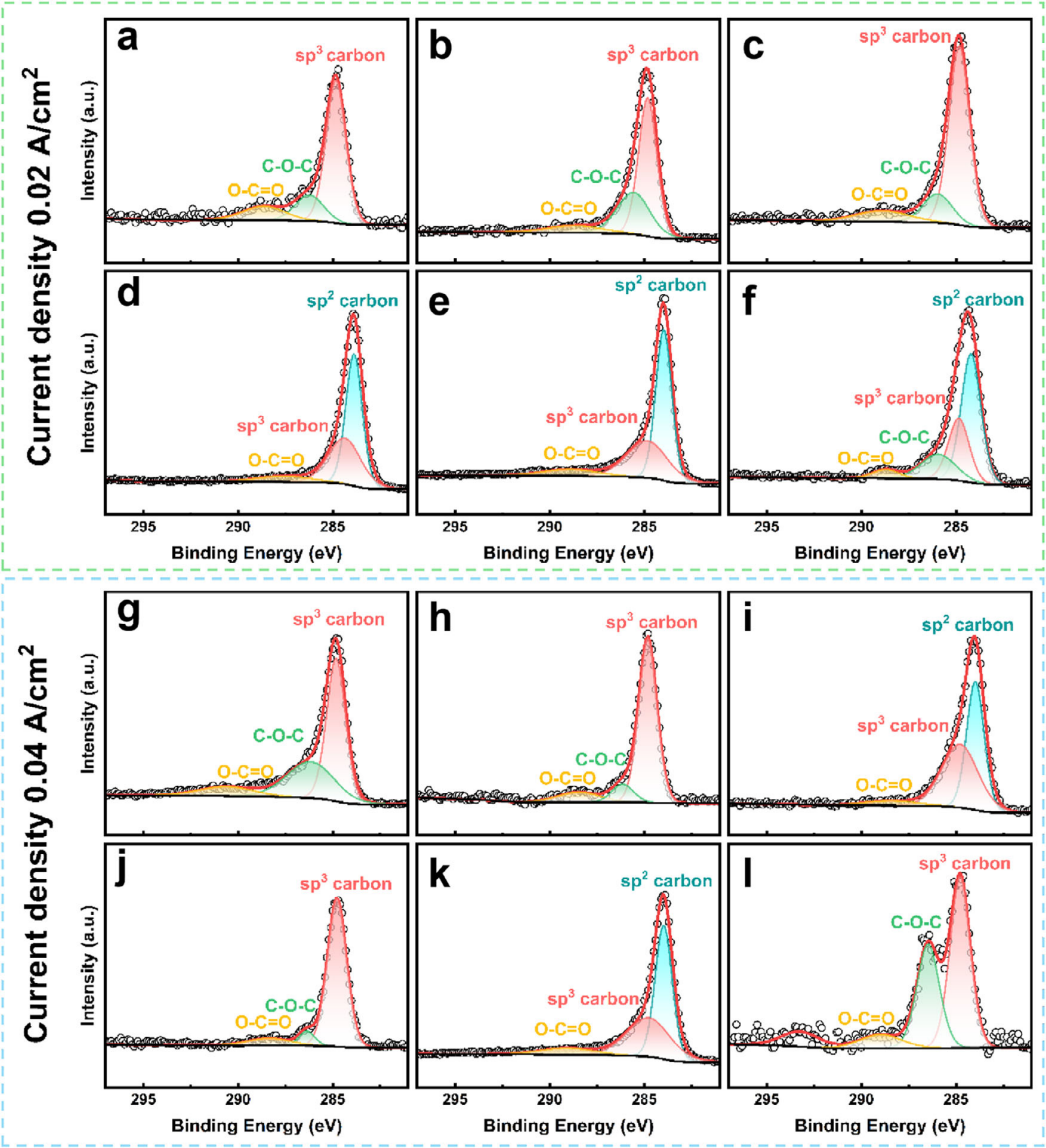
High‐resolution XPS C 1s spectra with peak fitting for samples prepared at a current density of 0.02 A/cm^2^ after instantaneous heat treatment at 500°C–900°C (a–e) and after linear‐ramp heat treatment at 700°C (f), and those prepared at 0.04 A/cm^2^ after instantaneous heat treatment at 500°C–900°C (g–k) and after linear‐ramp heat treatment at 700°C (l).

The thermal desorption of surface functional groups and the reconstruction of interfacial bonding directly govern the intrinsic mechanical strength and interfacial polarization of the composite. Based on XPS high‐resolution C 1s spectra, as presented in Figure 7, together with the peak‐fitted Ni 2p and O 1s spectra shown in Figures S7 and S8, this work separately deciphers the chemical state evolution kinetics of coatings prepared at LD and HD during heat treatment.

For the LD coating, the initial morphology was neuronal‐like with abundant microporosity, exposing numerous oxidation‐active sites at the CF substrate and coating defects. At 500°C–600°C, the O 1s spectra revealed that the interface was enriched with large quantities of polar groups such as C─O and C═O (Figure S8a,b), while the Ni 2p spectra, shown in Figure S7a,b, detected Ni─O oxidized states. Upon raising the temperature to 700°C, although a portion of NiO was reduced, as shown in Figure 7c,f and Figure S7c,f, the poor structural continuity precluded thorough chemical purification. Nevertheless, this discontinuous reduction process constructed a Ni/NiO/C heterogeneous multiphase interface between the carbon matrix and metallic Ni. The moderately retained polar oxygen‐containing groups and NiO phases, combined with the inherent microporosity of the coating, generated abundant nanoscale dielectric heterojunctions. Under an alternating electromagnetic field, these heterogeneous interfaces can act as strong centers for space charge polarization and dipole polarization, thereby substantially enhancing electromagnetic wave absorption loss from the chemical state perspective and rendering this process the pathway to achieving optimal shielding effectiveness. When the temperature was further elevated to 800°C–900°C, as shown in Figure 7d,e and Figure S7d,e, the coating underwent spheroidization, leading to extensive exposure of the carbon skeleton. Carbon etching reactions occurred in the presence of residual oxygen, resulting in chemical structural alteration and mechanical property degradation.

In contrast, the HD coating exhibited a distinctly different evolutionary trajectory by virtue of its dense and continuous physical barrier characteristics. During the initial heating stage at 500°C–600°C, as shown in Figure 7g,h and Figure S7g,h, the compact metallic layer effectively blocked the ingress of external residual oxygen and strictly confined the oxygen atoms introduced during pretreatment within a shallow solid‐solution interfacial region. After heating treatment at 700°C, the interfacial oxygen within the coating underwent chemical purification. Within this thermodynamic window, highly active carbon atoms at the interface engaged in an in situ redox reaction with NiO, generating a substantial quantity of elemental Ni. The C 1s spectra in Figure 7i,l, together with the Ni 2p spectra in Figure S7i,l, demonstrate that the total oxygen content of the system was reduced to a minimum level and the Ni─O component was almost entirely deoxidized and reduced to the metallic Ni state. Such interface bonding with a high proportion of the metallic state eliminates the steric hindrance and lattice distortion induced by heteroatoms, constructing a low‐defect, high‐purity Ni─C coherent physical interface. This provides the critical structural basis for enhancing the plastic toughness and load transfer efficiency of the composite, making this process the optimal route for achieving plastic toughness. Nanoscale cross‐sectional TEM‐EDS characterizations were performed on specimens treated under this processing condition, as illustrated in Figures S12 and S13. Compared with the continuous Ni/NiO/AC three‐phase interface in the as‐plated samples, after heat treatment at 700°C, the NiO phase significantly decreased, and the Ni/AC two‐phase interface became the dominant form. This elemental distribution pattern directly confirms that an interfacial redox reaction takes place between the NiO coating and the AC layer, driving microstructural reconstruction of the coating: the coating is predominantly transformed into the metallic Ni phase. In partial regions, oxygen is nearly eliminated, which is highly consistent with the attenuation of NiO characteristic peaks in XPS spectra. Meanwhile, local regions with trace residual oxygen remain at the interface and alternate with oxygen‐free domains. Such heterogeneous spatial distribution of oxygen constitutes the structural origin for the formation of Ni/NiO/C multiphase heterogeneous interfaces, which provide nanoscale material foundations for interfacial polarization and enhanced electromagnetic absorption loss. As the temperature was further raised to 800°C–900°C, excessive interfacial reactions and localized destruction of the carbon skeleton led to the re‐enrichment of oxygen‐containing impurities, a slight rebound of the oxygen signal, and a corresponding decrease in the proportion of metallic Ni.

### 2.5 MD Simulation

**FIGURE 8:**
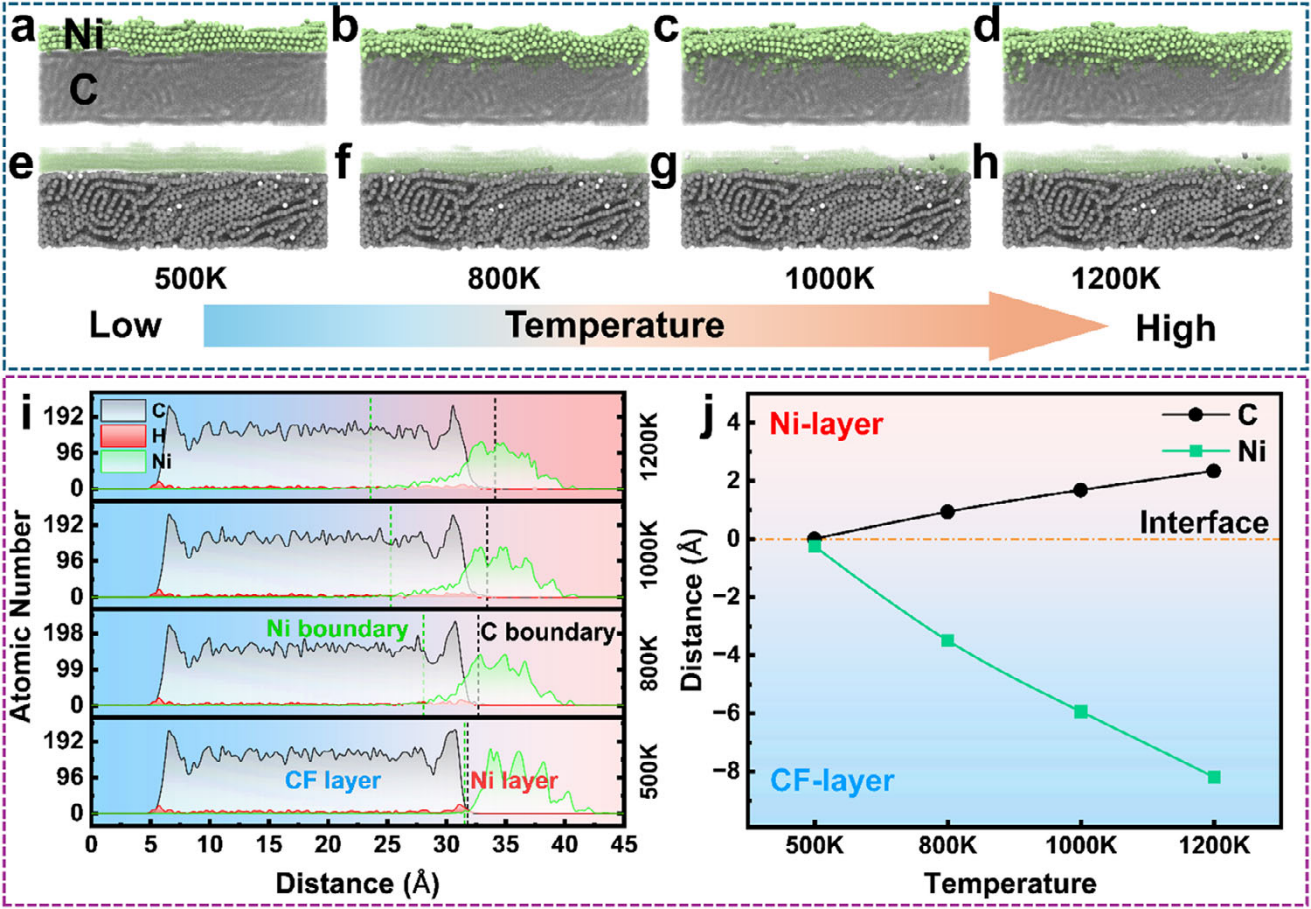
(a–h) Dynamic three‐dimensional atomic migration and penetration trajectories of the Ni─C interface under a temperature field driving force from 500 to 1200 K, respectively; (i) number density distribution profiles of Ni, C, and active H atoms extracted along the *y*‐axis perpendicular to the interface after simulation equilibration; (j) extracted evolution curves of the farthest diffusion positions of carbon and Ni atoms under different thermodynamic temperature plateaus.

To theoretically substantiate the macroscopic experimental observations and quantitatively characterize the atomic migration behavior, this study adopts the carbon modeling approach proposed by Joshi et al. (see Figure S9 for details) to construct an MD model that reflects the real physical topology of CF using LAMMPS [44, 45, 46]. The dynamic evolution of the Ni─C interface was systematically simulated over a temperature range of 500–1200 K, as presented in Figure 8. The simulation results achieved satisfactory cross‐scale consistency with the macroscopic morphological characterization: Figure 8a–h visually reproduces the three‐dimensional atomic migration trajectories with increasing temperature, while Figure 8i, displaying the atomic number density distribution perpendicular to the interface, and Figure 8j, showing the maximum diffusion distance as a function of temperature, collectively provide critical quantitative kinetic evidence for elucidating the interfacial bonding mechanism.

At the initial physical contact stage of 500 K (Figure 8a,e), Ni and C atoms only underwent small‐amplitude thermal vibrations in the vicinity of the interface. The number density distribution curves in Figure 8i reveal that the interface boundary between the Ni layer and the carbon substrate layer was extremely steep at this temperature, with virtually no overlapping region, indicating that the interface still retained its mechanical anchoring characteristics. When the temperature was raised to 800 K (Figure 8b,f), Ni atoms satisfied the diffusion activation energy barrier requirements and exhibited a certain degree of diffusion behavior into the interlayer structures and defect sites of the CF, demonstrating partial interfacial merging; nevertheless, the diffusion distance remained relatively short, and mechanical anchoring still dominated. As the temperature reached 1000 K, the atomic thermal kinetic energy overcame the diffusion activation energy barrier, and the interfacial nature underwent a fundamental transition (Figure 8c,g). The distribution curves at 1000 K show that the originally steep boundary of the carbon interface began to broaden, with a significant overlapping region emerging between the Ni and C number density distributions. Crucially, the active hydrogen atoms that had previously served to passivate the CF surface and defect edges underwent massive desorption under the catalytic and thermal driving of metallic Ni, with their density peak migrating distinctly toward the outer side of the coating. Hydrogen desorption not only greatly enhanced the interfacial bonding activity but also provided sufficient spatial conditions for the subsequent deep Ni─C interdiffusion. Upon entering the high‐temperature stage of 1200 K, atomic mixing became extremely intense (Figure 8d,h), and the overlapping region of elemental distributions expanded substantially, forming a gradient interdiffusion layer spanning several nanometers in width. Integrated with the quantitative analysis of the maximum diffusion distance in Figure 8j, this study reveals a significant asymmetric diffusion kinetic feature: as the temperature increases, the maximum diffusion distances of both Ni and C atoms exhibit a nearly linear increase; however, the diffusion depth and migration rate of Ni atoms are significantly greater than those of C atoms. At 1200 K, the deep penetration distance of Ni into the interior of the carbon matrix far exceeds the reverse diffusion distance of C into the top Ni layer.

This pronounced asymmetric diffusion primarily stems from the abundance of defects within the CF, including interlayer gaps, grain boundaries, and micropores. These irregular microstructures provide low‐energy‐barrier rapid diffusion channels for the smaller‐radius Ni atoms, thereby promoting an asymmetric cross‐interface diffusion mechanism dominated by the deep anchoring of Ni atoms into the carbon matrix and supplemented by the localized interfacial rearrangement of C atoms. At the nanoscale, this mechanism transforms the original physical contact interface into a chemically bonded transition zone, profoundly elucidating the microscopic mechanism of interfacial strengthening in Ni─CF composites at the atomic scale.

### 2.6 Mechanisms of Performance Improvement and Enhancement

This study aims to overcome the bottleneck that mechanical properties and EMI shielding effectiveness are difficult to synergistically enhance in conventional metallized CFs. Through broadband vector network analysis in the X‐band (8.2–12.4 GHz) and quasi‐static tensile testing, the EMI shielding and tensile properties of the materials under different processing conditions were systematically quantified, thereby revealing the synergistic enhancement mechanism and the performance trade‐off rule.

#### 2.6.1 Electromagnetic Shielding

The electrical transport properties of the coatings serve as the core physical foundation governing electromagnetic shielding effectiveness [47, 48]. As shown in Figure 2v–x, pristine CFs exhibit relatively high sheet resistance and resistivity due to their intrinsic semiconducting characteristics. After electrodeposition of Ni coatings, the sheet resistance and resistivity of the samples decrease significantly, while the electrical conductivity increases substantially. This confirms that metallization modification effectively constructs a continuous conductive network, which provides the fundamental prerequisite for electromagnetic shielding [49, 50].

With increasing heat treatment temperature, the electrical properties of the two types of coatings present distinct evolution behaviors. For the LD sample, electrical conductivity reaches a peak at 700°C, which is attributed to the healing of initial coating defects via interfacial atomic diffusion, thereby enhancing electrical continuity. When the temperature rises to 900°C, the spheroidization and fragmentation of the coating induce the breakdown of the conductive network, resulting in a marked decline in electrical conductivity. In contrast, the HD sample maintains lower sheet resistance and higher electrical conductivity across the entire temperature range by virtue of its initially dense coating structure. Furthermore, with rising temperature, grain boundary healing and grain growth further reduce electron scattering, leading to a continuous increase in electrical conductivity, which remains at an excellent level even at 900°C. This evolution pattern of electrical properties is highly consistent with the variation trend of the subsequent electromagnetic shielding performance, revealing the regulation mechanism of shielding performance from the perspective of electrical transport.

**FIGURE 9:**
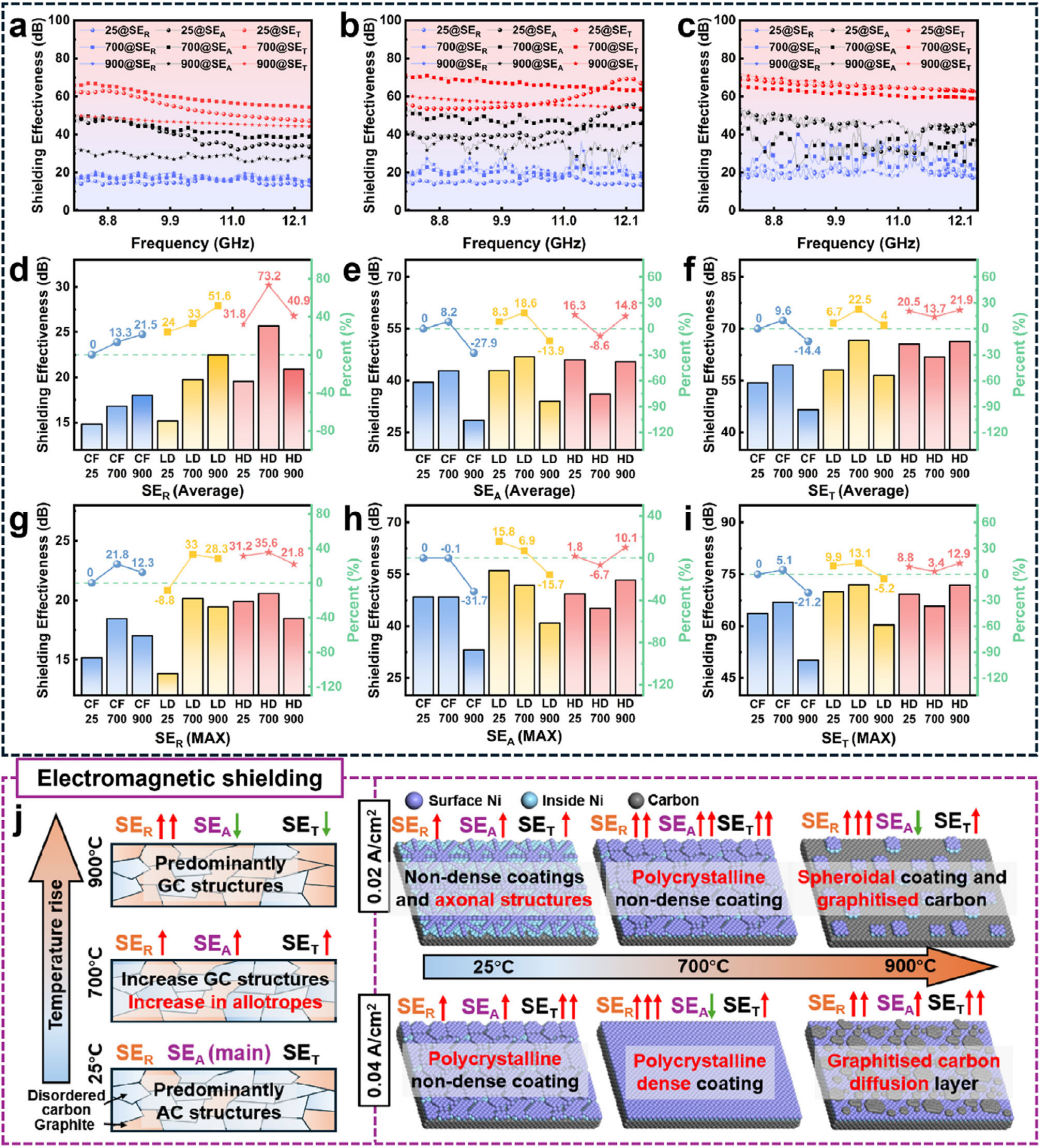
Broadband EMI shielding performance and loss mechanisms of the composites in the X‐band (8.2–12.4 GHz). Frequency‐domain curves in the X‐band for pristine CF (a), and those prepared at current densities of 0.02 A/cm^2^ (b) and 0.04 A/cm^2^ (c) at RT, 700°C, and 900°C; statistical comparison charts of the average (d–f) and maximum (g–i) values of SE_R_, SE_A_, and SE_T_ for samples subjected to different fabrication and heat treatment processes; (j) schematic illustration of the shielding mechanism accompanying the evolution of different interfacial phase structures.

Regarding EMI shielding performance, as shown in Figure 9, taking the pristine CF as the benchmark, its average reflection loss SE_R_, absorption loss SE_A_, and total shielding effectiveness SE_T_ were 14.83, 39.57, and 54.40 dB, respectively, with the corresponding maximum values reaching 15.16, 48.47, and 63.63 dB. After vacuum heat treatment at 700°C, the average SE_R_, SE_A_, and SE_T_ increased by 13.3%, 8.2%, and 9.6%, respectively, indicating that appropriate vacuum high‐temperature treatment alters the CF surface structure to a certain extent and confers improved EMI shielding performance. However, following vacuum heat treatment at 900°C, although the average SE_R_ exhibited a 21.5% enhancement, the high temperature severely compromised the absorption loss capability, with the average SE_A_ declining by 27.9% and the maximum SE_A_ dropping by 31.7%, leading to a 21.2% reduction in the maximum SE_T_.

Ni electroplating reshaped the shielding effectiveness landscape. The sample processed at LD with LH to 700°C exhibited the superior EMI shielding performance among all groups. Its average SE_R_, SE_A_, and SE_T_ were enhanced by 33.0%, 18.6%, and 22.5%, respectively, compared to the benchmark, while the maximum SE_R_, SE_A_, and SE_T_ were increased by 30.8%, 60.1%, and 21.8%, respectively, with the maximum SE_T_ attaining approximately 71.9 dB. However, upon raising the temperature to 900°C, severe spheroidization of the coating resulted in a dramatic 15.7% decline in the maximum SE_A_ and a 5.2% decrease in the maximum SE_T_. In contrast, the HD sample not only delivered a 13.7% enhancement in average SE_T_ and a remarkable 73.2% surge in average SE_R_ at 700°C, but also demonstrated exceptional service stability under the extreme thermal shock at 900°C: its average SE_R_ and SE_A_ did not deteriorate but instead further increased by 40.9% and 14.8%, respectively, with the average SE_T_ improving by 21.9% and the maximum SE_T_ reaching 71.8 dB, essentially on par with that of the LD 700°C sample.

Integrating the structural evolution model presented in Figure 9j, the shielding response can be rationalized as follows. At RT, shielding is predominantly governed by absorption loss, originating from the disordered carbon and the initial polycrystalline Ni structure. At 700°C, the coarsening of graphitic microcrystalline within the carbon matrix intensifies polarization loss, while the distinctive polycrystalline non‐dense coating of the LD sample preserves moderate microporosity and abundant heterogeneous interfaces, thereby substantially enhancing multiple reflections and interfacial polarization of electromagnetic waves and yielding the highest SE_A_ and total shielding effectiveness. At 900°C, the interface undergoes further graphitization; nevertheless, for the LD sample, the irreversible spheroidization of the coating disrupts the conductive network, causing a precipitous drop in absorption loss. Conversely, the HD sample, by virtue of its initially dense structure, evolves a continuous graphitized carbon diffusion layer, which not only stabilizes the macroscopic conductive framework but also strengthens the localized electromagnetic response, thereby ensuring sustained shielding performance stability under extremely high temperatures.

#### 2.6.2 Mechanical Properties

**FIGURE 10:**
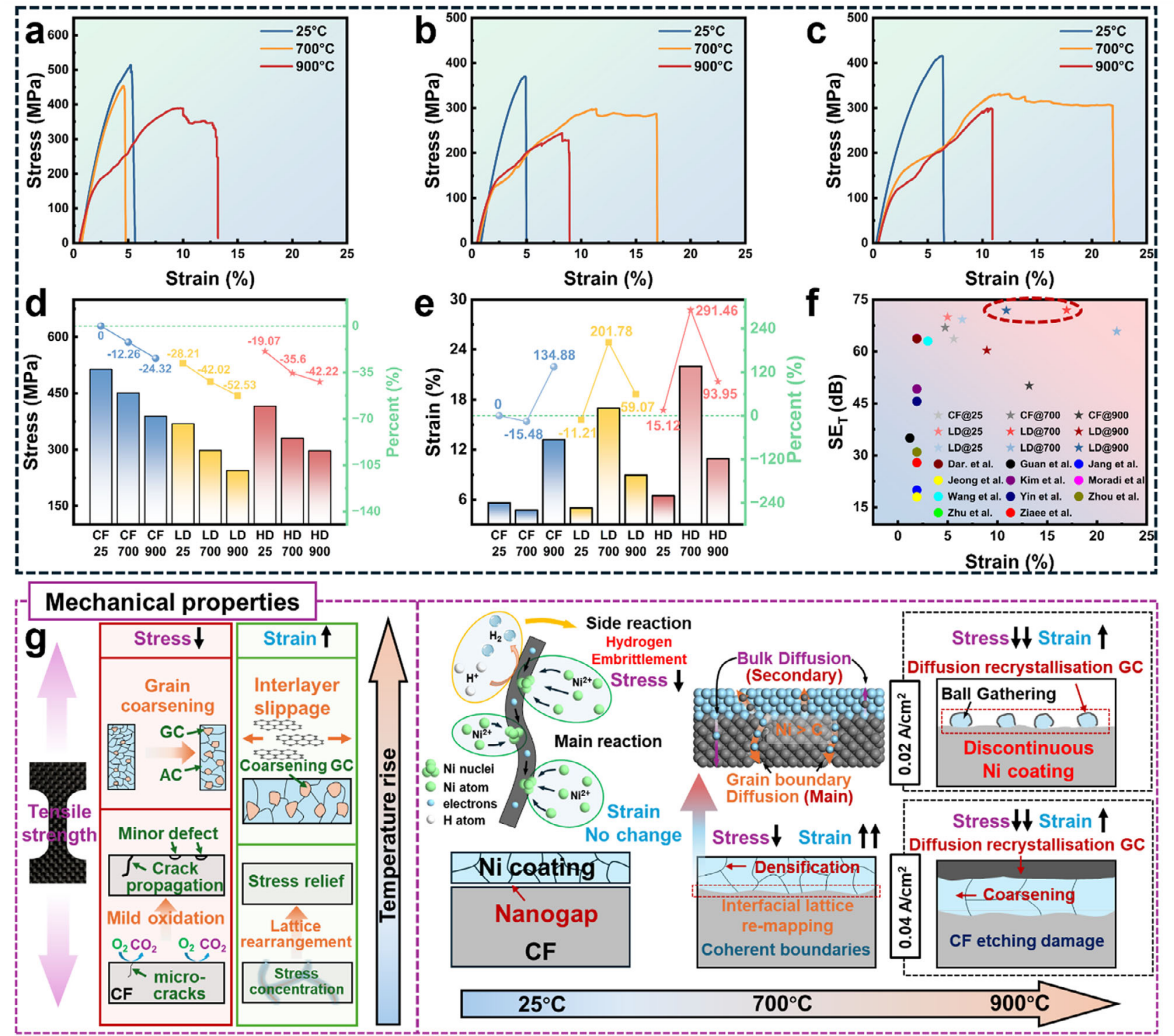
Quasi‐static tensile stress–strain response curves of pristine CFRP (a), CFRP with LD electroplating (b), and CFRP with HD electroplating (c) at RT, 700°C, and 900°C, along with the corresponding statistical histograms of maximum tensile strength (d) and elongation at break (e); (f) two‐dimensional performance comparison chart of total EMI shielding effectiveness versus maximum fracture strain, illustrating the application orientation of different process combinations and the previously reported carbon fiber‐based EMI shielding materials [35, 50, 51, 52, 53, 54, 55, 56, 57, 58, 59]; (g) mechanism model of the micromechanical failure behavior evolution.

In terms of tensile mechanical behavior, as shown in Figure 10a–e, the pristine CFRP composite exhibited typical brittle fracture characteristics, with a maximum fracture strain of 5.62% and a maximum tensile strength of 514 MPa. High‐temperature heat treatment induced an opposite evolution trend between strength and toughness: the fracture strain increased to 13.2%, corresponding to a relative enhancement of 134.88%, whereas the tensile strength decreased to 389 MPa. Although LD Ni modification imparted significant toughening, it incurred an strength penalty. Under heat treatment at 700°C, the maximum fracture strain increased to 16.96% (a relative enhancement of 201.78%), but the tensile strength dropped to 298 MPa. When the temperature was further raised to 900°C, the fracture strain declined to 8.94%, and the strength was merely 244 MPa, indicating an overall deterioration in mechanical properties.

In contrast, HD Ni modification fundamentally altered the failure mode of the CF composite and broadened its mechanical boundaries. At a heat treatment temperature of 700°C, the stress–strain curve displayed an extensive plastic deformation plateau, and the maximum fracture strain reached 22.0% — a 291.46% enhancement over the pristine composite — thereby achieving a transition from brittle fracture to plastic and ductile fracture. Notably, the maximum tensile strength was maintained at 331 MPa, yielding a strength retention ratio considerably higher than that of the LD counterpart. According to the strengthening‐toughening and failure mechanism analysis presented in Figure 10g, the reduction in tensile strength fundamentally arises from the combined effects of high‐temperature grain coarsening, interfacial microcrack propagation, and mild oxidation/carbon etching damage, whereas the remarkable improvement in strain capability primarily originates from interlayer slippage, stress relaxation, and lattice rearrangement. For the HD sample heat‐treated at 700°C, the continuous and dense Ni layer underwent densification and interfacial lattice remapping driven by thermodynamic forces, forming a coherent interfacial transition zone that, in synergy with the nano‐equiaxed Ni grain network, buffered and dispersed local stress, thereby achieving a multiple‐fold increase in plastic strain at the expense of only a moderate sacrifice in strength. Conversely, the mechanical degradation of the LD sample heat‐treated at 900°C is attributed to high‐temperature agglomeration and spheroidization of the Ni coating and the resulting loss of interfacial continuity.

A horizontal performance benchmarking comparison between the core performance metrics of this work and previously reported carbon fiber‐based EMI shielding composites is illustrated in Figure 10f. The results demonstrate a ubiquitous trade‐off between EMI shielding capacity and mechanical toughness in conventional modification systems. Metallized coating modification can dramatically boost electrical conductivity and shielding effectiveness, yet it readily introduces brittle phases and stress concentration at the fiber‐matrix interface, consequently decreasing the fracture elongation of composites. In contrast, sole interfacial toughening strategies generally sacrifice continuous conductive networks, failing to realize high‐efficiency EMI shielding concurrently. The interfacial modification strategy proposed herein, which integrates electrodeposition with LH vacuum heat treatment, successfully overcomes the performance trade‐off limitation. The LD specimen treated at 700°C delivers a total shielding effectiveness of 71.9 dB in the X band while retaining a fracture elongation exceeding 16%. For the HD counterpart subjected to 700°C treatment, the fracture elongation is further elevated to 22% with shielding effectiveness maintained at nearly 70 dB. The integrated performance of both samples stands at an advanced level among comparable research, which validates the remarkable advantages of the proposed strategy in synergistically enhancing mechanical and EMI shielding performances.

On this basis, the performance characteristics of diverse processing routes can be further analyzed to formulate targeted process selection criteria for differentiated service conditions. The LD@700°C process, leveraging the multiple reflections and polarization absorption mechanisms triggered by the unique polycrystalline non‐dense diffusion structure, achieves ultimate EMI shielding effectiveness while retaining moderate plasticity, and thus represents the preferred route for realizing extreme shielding protection. The HD@700°C process, by constructing a highly uniform coherent Ni─C bonded interface, delivers a 13.7% enhancement in shielding effectiveness while simultaneously attaining the highest fracture strain and favorable strength retention, making it the optimal choice for meeting the demands of high deformation tolerance, strong load‐bearing capacity, and stable shielding performance.

## 3 Conclusions

To address the inherent trade‐off dilemma between EMI shielding effectiveness and mechanical ductility in conventional metallized CF composites, this work proposes a strategy to construct neuronal‐like Ni coatings via LD electrodeposition and tune interfacial microstructures by LH vacuum heat treatment. Experimental results verify that LH at 700°C is the optimal processing condition for optimized macroscopic performance. Such thermal treatment triggers morphological reconstruction and interfacial lattice rearrangement, fundamentally overcoming the performance trade‐off between EMI shielding capacity and mechanical ductility, and clarifies the structure‐property relationship governing the coordinated enhancement of two macroscopic properties driven by interfacial microstructural evolution.

Coating microstructural evolution is co‐determined by electrodeposition current density and heat treatment temperature. After LH at 700°C, the neuronal‐like coatings fabricated at LD evolve into micro‐nano porous network architectures, accompanied by the in situ formation of Ni/NiO/C multiphase heterogeneous structures and vertically embedded lattice interlocking configurations at the interfaces. By contrast, excessive thermal treatment at 900°C causes coating spheroidization and disrupts the continuous conductive skeleton. Dense coatings deposited at HD exhibit favorable thermal stability yet contain no porous interfaces for energy dissipation. The synergistic effect of micro‐nano porous morphology and heterogeneous interfaces creates abundant loss pathways for electromagnetic waves via multiple scattering, reflection, and interfacial polarization. As a result, the optimized specimen with neuronal‐like coatings achieves a total shielding effectiveness of 71.9 dB across the X band, outperforming as‐deposited samples and all other control groups.

In terms of mechanical properties, the interfacial bonding and mechanical interlocking formed during 700°C LH eliminate the incoherent brittleness of conventional metallized interfaces, elevating interfacial load transfer efficiency and fracture energy dissipation capacity. The composite reinforced with optimized neuronal‐like coatings possesses a fracture elongation exceeding 16%. Benchmark comparisons with existing literature demonstrate that the proposed route realizes simultaneous improvement in ductility while retaining outstanding EMI shielding performance, with its comprehensive properties ranking at the cutting edge of analogous research. This study provides a solid theoretical and experimental foundation for interfacial engineering and structural design of advanced multifunctional EMI shielding composites.

## 4 Experimental Methods

### 4.1 Materials and Chemical Reagents

The substrate employed in this study was a polyacrylonitrile (PAN)‐based T300‐grade 3K carbon fiber plain weave fabric (precursor fiber supplied by Toray, Japan; fabric woven by Zhongfu Carbon Fiber Products Co., Ltd.). The polymer matrix consisted of 4674‐type epoxy resin and its corresponding H‐4073‐3 curing agent (YUTAO Company). The chemical reagents used included concentrated nitric acid (HNO_3_, 68 wt%), acetone, Ni sulfate hexahydrate (NiSO_4_·6H_2_O), dilute sulfuric acid, Ni chloride (NiCl_2_), boric acid (H_3_BO_3_), and sodium dodecyl sulfate (SDS). All reagents were of analytical grade (AR) and obtained from Shanghai Aladdin Reagent Co., Ltd.

### 4.2 Interfacial Activation of Carbon Fiber Plain Weave Fabric

The tailored carbon fiber fabric was immersed in acetone for 8 h to remove the commercial sizing agent. After repeated suction filtration washing with deionized water, the fabric was dried in a vacuum oven at 60°C for 8 h. Subsequently, the dried sample was immersed in concentrated HNO_3_ solution for 6 h of liquid‐phase oxidative etching to introduce abundant polar oxygen‐containing adsorption sites, such as ─OH and ─COOH, onto the carbon skeleton surface. After etching, the fabric was rinsed with deionized water until the washing became neutral, followed by vacuum drying at 60°C for another 8 h before use.

### 4.3 Watts Bath Electrodeposition and Vacuum Metallurgical Heat Treatment

A Watts Ni plating process was adopted. The electrolyte composition was: 100 g/L NiSO_4_·6H_2_O, 40 g/L NiCl_2_, 30 g/L H_3_BO_3_, and 0.05 g/L SDS, with the pH maintained at 3.5 ± 0.2. To homogenize the electric field distribution, the edges of the activated carbon fiber cloth (dimensions 6 cm × 7 cm) were wrapped with conductive aluminum foil (see Figure S9). Electrodeposition was carried out at constant areal current densities of 0.02 A/cm^2^ and 0.04 A/cm^2^ for 10–30 min to obtain samples with varying coating thicknesses.  ° After deposition, the as‐fabricated Ni/CF samples were thoroughly rinsed with deionized water and dried, then loaded into a quartz tube mounted on a high‐temperature tube furnace, and the system was evacuated to a high vacuum below 5 × 10^−3^ Pa prior to heat treatment. For the IH control groups, the quartz tube containing samples was rapidly inserted into the isothermal zone of the furnace once the furnace temperature was preheated to 500°C, 600°C, 700°C, 800°C and 900°C respectively, followed by 2 h of isothermal heat treatment to induce interfacial reconstruction. For the LH group, the quartz tube was placed in the furnace chamber at room temperature, and the temperature was programmatically increased to 700°C at a linear ramping rate of 5°C/min, followed by 2 h of isothermal holding.

### 4.4 Hot‐Press Curing and Molding of Composite Materials

The modified Ni/CF fabric was thoroughly impregnated with a mixed solution of epoxy resin and curing agent. The resulting prepreg was laid up and placed into a standard autoclave. Under a vacuum of −0.1 MPa and a molding pressure of 75 kgf/cm^2^, the system was held at a constant temperature of 70°C for 3 h to ensure complete crosslinking and curing of the resin and the elimination of micro voids.

### 4.5 Multi‐Scale Characterization and Performance Testing

A TESCAN CLARA field‐emission SEM equipped with an EDS was used to observe the three‐dimensional surface morphology, measure the coating thickness, and analyze the elemental distribution. Cross‐sectional TEM specimens at the CF/Ni coating interface were fabricated using a Helios 5 CX FIB system. Before specimen preparation, a tungsten protective layer was deposited on the target observation region to prevent structural damage to the coating and near‐surface interface induced by ion beam bombardment. Subsequent rough cutting and stepwise thinning were performed with high‐energy Ga^+^ ion beams, followed by fine polishing with low‐energy ion beams to obtain electron‐transparent cross‐sections thinner than 100 nm, which maximally preserved the pristine interfacial microstructure. Multi‐scale characterizations of interfacial microstructure and elemental distribution were carried out via two high‐resolution field‐emission TEM instruments, including JEOL JEM‐F200 and Tecnai G2 F30. Bright‐field (BF), high‐angle annular dark‐field (HAADF), and HRTEM micrographs were acquired, while EDS was coupled to map elemental distribution across the interface. These characterizations visually revealed the evolution of interfacial phase constitution and elemental diffusion behavior before and after heat treatment. Furthermore, two‐dimensional strain tensor distributions within interfacial regions were calculated from HRTEM lattice images by GPA, enabling quantitative evaluation of lattice misfit and stress relaxation at interfaces upon thermal treatment. A Rigaku MiniFlex600 X‐ray diffractometer (XRD, Cu Kα radiation) and a LabRAM Odyssey Reflex Raman spectrometer (532 nm excitation wavelength) were employed to analyze the lattice structure and defect states. A Thermo Scientific Escalab 250XiUSA XPS was utilized for detailed fitting of the interfacial bonding states. The surface sheet resistance of CF fabrics modified by different processes was quantitatively measured using a CXT 3565 four‐point probe tester based on the direct‐current (DC) four‐point probe method. Before testing, the samples were cut into regular specimens measuring 30 mm × 30 mm, ensuring that the coating surfaces were flat, wrinkle‐free, and free of mechanical damage. During the measurement, the four probes were brought into stable ohmic contact with the sample surface to eliminate the interference of contact resistance on the test results. For each sample, parallel measurements were conducted on five randomly selected non‐overlapping regions, and the arithmetic mean was taken as the final sheet resistance value of the sample to mitigate test errors arising from local inhomogeneity of the coating. Quasi‐static tensile tests on the cured panels were performed using an Instron 34TM‐50 universal testing machine to obtain the fracture mechanical characteristics. Broadband EMI shielding effectiveness and associated loss parameters were measured with a Keysight E5071C vector network analyzer (VNA) equipped with a coaxial test fixture.

### 4.6 MD Modeling and Simulation

Following the CF modeling paradigm proposed by Joshi et al. [10, 60]. (detailed modeling workflow shown in Figure S9), a turbostratic carbon structure with realistic physical topology was constructed using LAMMPS. Three types of three‐layer graphene microcrystallites with in‐plane dimensions of 11.8, 24.1, and 43.8 Å were selected as fundamental building units, with their edges passivated by hydrogen atoms. 15 microcrystallites of each size were randomly packed into a supercell using the Packmol program [61]. After energy minimization, the system was compressed to the target density (1.9 g/cm^3^) under periodic/non‐periodic boundary constraints, resulting in a supercell size of 110 × 27 × 50 Å^3^. Free hydrogen atoms were removed to induce chemical crosslinking, and the remaining dangling bonds were subsequently re‐passivated with hydrogen to stabilize the system. A 50 Å vacuum layer was introduced above the substrate, and a vapor deposition zone with a thickness of 10 Å was defined at a distance of 20 Å from the surface along the positive y‐axis. A total of 2000 Ni atoms were deposited onto the carbon surface at a rate of 100 atoms/fs. Following energy minimization of the entire system, a gradient annealing relaxation was performed in the NVT ensemble: the temperature was progressively increased from 500 to 1200 K with a step size of 100 K, and isothermal relaxation was conducted for 1 ns at each temperature plateau to extract the kinetic evolution trajectory of Ni─C cross‐interface interdiffusion.

## Author Contributions

**Ben Jia**: conceptualization, methodology, writing – review and editing, visualization, investigation. **Wei Cheng**: conceptualization, writing – original draft, writing – review and editing, investigation, validation, software. **Biao Chen**: conceptualization, validation, methodology. **Heyuan Huang**: project administration, funding acquisition, writing – review and editing, resources, data curation. **Yongjun Zhang**: writing – review and editing, methodology. **Shuhan Xiang**: investigation, visualization, software. **Xiaopeng Wan**: conceptualization, supervision. **Zhuo Liu**: validation, formal analysis, supervision, funding acquisition.

## Funding

This work was supported by the National Natural Science Foundation of China (Grant Nos. 12141203 and 82272155); the National Science Foundation for Young Scientists of China (Grant No. 52405516); the Fundamental Research Funds for the Central Universities (Grant No. D5000230052); the Shaanxi Innovation Ability Support Plan Project Funds (Grant No. 2024RS‐CXTD‐29); Young Elite Scientists Sponsorship Program by CAST (Ph.D. Fellowship Program: 156‐O‐430‐0000867‐9).

## Conflicts of Interest

The authors declare no conflicts of interest.

## Supporting information




**Supporting File**: advs77333‐sup‐0001‐SuppMat.docx.

## Data Availability

The data that support the findings of this study are available from the corresponding author upon reasonable request.

## References

[1] C. W. Lahive , S. H. Dempsey , S. E. Reiber , et al., “Acetolysis for Epoxy‐Amine Carbon Fibre‐Reinforced Polymer Recycling,” Nature 642, no. 8068 (2025): 605–612, 10.1038/s41586-025-09067-y. 40468077

[2] J. Y. Choi and S.‐H. Ahn , “Mesoscale Carbon Fiber Lattices with Foam‐Like Weight and Bulk Strength,” Nature Communications 17, no. 1 (2026): 3615, 10.1038/s41467-026-72105-4. PMC13100039 42014694

[3] H. Wang , S. Huo , V. Chevali , et al., “Carbon Fiber Reinforced Thermoplastics: from Materials to Manufacturing and Applications,” Advanced Materials 37, no. 27 (2025): 2418709, 10.1002/adma.202418709. 40263922 PMC12243730

[4] G. Kang , G. Kwon , J. Jeon , et al., “Electromagnetic Interference Shielding Using Metal and MXene Thin Films,” Nature 647, no. 8089 (2025): 356–363, 10.1038/s41586-025-09699-0. 41162708

[5] P. Rani and V. R. K. Murthy , “Development of MXene Based Organic Polymer Nanocomposites as Highly Efficient Electromagnetic Interference Shielding Materials: a Review,” Coordination Chemistry Reviews 548 (2026): 217104, 10.1016/j.ccr.2025.217104.

[6] L. Wang , W. Ren , X. Hu , et al., “Biomass Materials and Their Derivatives for Electromagnetic Interference Shielding: a Review,” Journal of Materials Science & Technology 243 (2026): 28–44, 10.1016/j.jmst.2025.04.019.

[7] X. Hou , X.‐R. Feng , K. Jiang , Y.‐C. Zheng , J.‐T. Liu , and M. Wang , “Recent Progress in Smart Electromagnetic Interference Shielding Materials,” Journal of Materials Science & Technology 186 (2024): 256–271, 10.1016/j.jmst.2024.01.008.

[8] W. Xie , Y. Yan , M. Tian , et al., “Extremely Durable Integrated Metallic Micromesh for Multifunctional Transparent Electromagnetic Interference Shielding,” Sustainable MaterTechnol 47 (2026): e01844, 10.1016/j.susmat.2025.e01844.

[9] L. Yao , K. Zheng , N. Koripally , et al., “Structural Pseudocapacitors with Reinforced Interfaces to Increase Multifunctional Efficiency,” Science Advances 9, no. 25 (2023): adh0069, 10.1126/sciadv.adh0069. PMC10289652 37352340

[10] Z. Li , W. Chen , D. Seveno , and D. Jiang , “Modeling and Mechanism of the Mechanical Interlocking for the Carbon fiber/Epoxy Interphase,” Carbon 233 (2025): 119861, 10.1016/j.carbon.2024.119861.

[11] J. Sun , F. Zhao , Y. Yao , Z. Jin , X. Liu , and Y. Huang , “High Efficient and Continuous Surface Modification of Carbon Fibers with Improved Tensile Strength and Interfacial Adhesion,” Applied Surface Science 412 (2017): 424–435, 10.1016/j.apsusc.2017.03.279.

[12] J. Moosburger‐Will , E. Lachner , M. Löffler , et al., “Adhesion of Carbon Fibers to Amine Hardened Epoxy Resin: Influence of Ammonia Plasma Functionalization of Carbon Fibers,” Applied Surface Science 453 (2018): 141–152, 10.1016/j.apsusc.2018.05.057.

[13] D. Gravis , S. Moisan , and F. Poncin‐Epaillard , “Characterization of Surface Physico‐Chemistry and Morphology of Plasma‐Sized Carbon fiber,” Thin Solid Films 721 (2021): 138555, 10.1016/j.tsf.2021.138555.

[14] M. Lilli , M. Jurko , V. Sirjovova , et al., “Basalt Fibre Surface Modification via Plasma Polymerization of Tetravinylsilane/Oxygen Mixtures for Improved Interfacial Adhesion with Unsaturated Polyester Matrix,” Materials Chemistry and Physics 274 (2021): 125106, 10.1016/j.matchemphys.2021.125106.

[15] D. J. Eyckens , K. Jarvis , A. J. Barlow , et al., “Improving the Effects of Plasma Polymerization on Carbon fiber Using a Surface Modification Pretreatment,” Composites Part A: Applied Science and Manufacturing 143 (2021): 106319, 10.1016/j.compositesa.2021.106319.

[16] H. Qin , H. Zhou , W. Guo , F. Yan , and H. Xiao , “Reversal of Wettability of Carbon Cloth by Microwave‐Assisted Modification Technology for Efficient Oil‐Water Separation Application,” Surface and Coatings Technology 419 (2021): 127260, 10.1016/j.surfcoat.2021.127260.

[17] X. Liu , J. Luo , J. Fan , et al., “Comprehensive Enhancement in Overall Properties of MWCNTs‐COOH/Epoxy Composites by Microwave: an Efficient Approach to Strengthen Interfacial Bonding via Localized Superheating Effect,” Composites Part B: Engineering 174 (2019): 106909, 10.1016/j.compositesb.2019.106909.

[18] H. Pu , W. He , C. Yan , et al., “Enhancing the Interfacial and Electromagnetic Shielding Properties of CF/EP Composites via Fe‐Based MOFs in Situ Pyrolysis,” Applied Surface Science 733 (2026): 166622, 10.1016/j.apsusc.2026.166622.

[19] S. Ayyagari , M. Al‐Haik , Y. Ren , et al., “Metal Organic Frameworks Modification of Carbon fiber Composite Interface,” Composites Part B: Engineering 224 (2021): 109197, 10.1016/j.compositesb.2021.109197.

[20] H. Moradi , K. Nasouri , and G. Askari , “Effect of Morphology and Microstructure of NiCo Nanoparticles on the Electromagnetic Shielding Behavior of Flexible and Durable NiCo‐Coated Carbon Fibers,” Journal of Materials Science: Materials in Electronics 35, no. 1 (2024): 89, 10.1007/s10854-023-11878-6.

[21] J. Zhang , J. Zheng , X. Chen , Q. Liao , and T. Li , “Study on Ni/P‐Coating Deposition and Interface Behavior in Solid‐Solid/Solid‐Liquid Compositing Process,” Journal of Materials Research and Technology 30 (2024): 7998–8005, 10.1016/j.jmrt.2024.05.180.

[22] W. Wang , Z. Zhai , J. Liu , Y. Wang , and Z. Yang , “Influence of Carbon fiber Nickel Electroplating on the Electromagnetic Interference Shielding and Mechanical Properties of Carbon fiber Reinforced Polyamide 6 Composites,” Polymer Composites 44, no. 12 (2023): 8838–8848, 10.1002/pc.27741.

[23] S. Zhu , S. Yan , Y. Gao , et al., “Enhancing the Electromagnetic Shielding and Mechanical Properties of CF/PEEK Composites via Low‐Concentration Fluff and Ni‐Co Alloy Plating,” Thin‐Walled Structures 205 (2024): 112563, 10.1016/j.tws.2024.112563.

[24] C. Chen , J. Guan , N. W. Li , et al., “Lotus‐Root‐like Carbon Fibers Embedded with Ni–Co Nanoparticles for Dendrite‐Free Lithium Metal Anodes,” Advanced Materials 33, no. 24 (2021): 2100608, 10.1002/adma.202100608. 33960042

[25] Y. Zhang , H. Si , H. Liu , et al., “Heterogeneous and Hierarchical ni/C/SiO_2_ Composite with Tunable Electromagnetic Wave Absorption,” Materials Today Physics 36 (2023): 101149, 10.1016/j.mtphys.2023.101149.

[26] Y. Qiao , Y. Wang , J. Yang , Q. Li , and J. Gu , “High Performance Porous Ni@Cf Paper with Excellent Electromagnetic Shielding Properties,” Composites Part A: Applied Science and Manufacturing 172 (2023): 107618, 10.1016/j.compositesa.2023.107618.

[27] Y. Liu , X. Xiao , H. Liu , et al., “Ultra‐Broadband Microwave Absorbers via Gradient EM Parameters and Engineered Nanogaps in 1D NiO/Ni/Gap/C Composites,” ACS Applied Electronic Materials 7, no. 18 (2025): 8598–8608, 10.1021/acsaelm.5c01389.

[28] S. Wang , Y. Liu , J. Yang , and X. Su , “Microwave Absorption Properties of Flexible Fabric Coating Containing Ni Decorated Carbon fiber with Frequency Selection Surface Incorporation,” Diamond and Related Materials 143 (2024): 110872, 10.1016/j.diamond.2024.110872.

[29] Y. Hu , M. Jiang , X. Cong , G. Liu , X. Yi , and X. Liu , “Lightweight, Multifunctional Recycled Carbon Fibre/MXene/PEDOT: PSS Nonwoven Veils with Double‐Layered Structure for Excellent Electromagnetic Interference Shielding,” Chemical Engineering Journal 489 (2024): 151122, 10.1016/j.cej.2024.151122.

[30] M. Song , Z. Liu , Y. Wang , et al., “Industrial‐Grade Flexible Carbon Fiber Paper/MXene Composite Electromagnetic Shielding Material with Ultra‐Large Area and Ultra‐High Performance,” Advanced Functional Materials 35, no. 27 (2025): 2421422, 10.1002/adfm.202421422.

[31] Y. Hu , S. Pang , J. Li , J. Jiang , and D. G. Papageorgiou , “Enhanced Interfacial Properties of Hierarchical MXene/CF Composites via Low Content Electrophoretic Deposition,” Composites Part B: Engineering 237 (2022): 109871, 10.1016/j.compositesb.2022.109871.

[32] Y. Hu , G. Yang , J. Chen , et al., “Interfacial Engineering of Hybrid MXene‐Ni‐CF Tri‐Core–Shell Composites for Electromagnetic Interference Shielding and E‐Heating Applications,” Composites Part A: Applied Science and Manufacturing 178 (2024): 107990, 10.1016/j.compositesa.2023.107990.

[33] Q. Li , Y. Sun , G. Li , X. Yang , and X. Zuo , “Enhancing Interfacial and Electromagnetic Interference Shielding Properties of Carbon fiber Composites via the Hierarchical Assembly of the MWNT/MOF Interphase,” Langmuir 38, no. 46 (2022): 14277–14289, 10.1021/acs.langmuir.2c02344. 36351284

[34] P. K. Gangineni , S. Yandrapu , S. K. Ghosh , A. Anand , R. K. Prusty , and B. C. Ray , “Mechanical Behavior of Graphene Decorated Carbon fiber Reinforced Polymer Composites: an Assessment of the Influence of Functional Groups,” Composites Part A: Applied Science and Manufacturing 122 (2019): 36–44, 10.1016/j.compositesa.2019.04.017.

[35] S. Zhu , R. Shi , M. Qu , et al., “Simultaneously Improved Mechanical and Electromagnetic Interference Shielding Properties of Carbon fiber Fabrics/Epoxy Composites via Interface Engineering,” Composites Science and Technology 207 (2021): 108696, 10.1016/j.compscitech.2021.108696.

[36] J. Ma , H. Ma , S. Zhang , L. Han , H. Gao , and D. Wen , “Boosting Electrothermal Conversion Coefficient of Carbon Fibers for De‐Icing Applications through Ni‐Cu Co‐Deposition on Carbon Fibers,” Applied Thermal Engineering 298 (2026): 130901, 10.1016/j.applthermaleng.2026.130901.

[37] Z. Liu , W. Cheng , D. Mu , Q. Lin , X. Xu , and H. Huang , “Growth Mechanisms of Interfacial Carbides in Solid‐State Reaction between Single‐Crystal Diamond and Chromium,” Journal of Materials Science & Technology 144 (2023): 138–149, 10.1016/j.jmst.2022.10.022.

[38] W. Cheng , Z. Liu , Q. Lin , et al., “Towards Tailorable Interface Microstructure through Solid‐State Interface Reaction between Synthetic Diamond Grits and Sputtered Ni‐Cr Binary Alloy,” Applied Surface Science 596 (2022): 153531, 10.1016/j.apsusc.2022.153531.

[39] L. Shi , M. Sessim , M. R. Tonks , and S. R. Phillpot , “High‐Temperature Oxidation of Carbon fiber and Char by Molecular Dynamics Simulation,” Carbon 185 (2021): 449–463, 10.1016/j.carbon.2021.09.038.

[40] R. Chen , Z. Yu , K. Qiao , et al., “Achieving Strong Corrosion Protection for Magnetic Refrigeration Materials via Electroplated Nickel Coatings,” Advanced Functional Materials 36, no. 27 (2026): 26039, 10.1002/adfm.202526039.

[41] W. Zhang , B. He , J. Shang , et al., “Novel Cu_2_O(SO_4_)@NiO Nanocomposite as Peroxymonosulfate Activator for Effective Degradation of Ciprofloxacin,” Chemical Engineering Journal 506 (2025): 160316, 10.1016/j.cej.2025.160316.

[42] S. Sharafzadeh , J. Zolgharnein , A. Nezamzadeh‐Ejhieh , and S. Dermanaki Farahani , “Co‐Precipitation Synthesis and Characterization of Zn(II)/Ni(II) Magnetic Heterostructure Ferrite for Photocatalytic Degradation of Metronidazole,” Surfaces and Interfaces 59 (2025): 105917, 10.1016/j.surfin.2025.105917.

[43] H. Ou‐Yang , X. Zhou , Y. Zhang , et al., “Interface Enhancement of 3D Printed CF/PLA Composites by Introducing “Rigid‐Flexible” Structures through Multi‐Bond Synergies,” Composite Structures 372 (2025): 119613, 10.1016/j.compstruct.2025.119613.

[44] M. Yao and X. Xiao , “Advance of Molecular Dynamics Simulations in the Thermophysical Properties of Molten Salts: a Critical Review,” Renewable and Sustainable Energy Reviews 229 (2026): 116609, 10.1016/j.rser.2025.116609.

[45] F. Birks , M. Nutter , T. D. Swinburne , and J. R. Kermode , “Efficient and Accurate Spatial Mixing of Machine Learned Interatomic Potentials for Materials Science,” npj Computational Materials 12, no. 1 (2026): 110, 10.1038/s41524-026-01982-6. 41836470 PMC12987727

[46] H. Zhou , M. Zhang , L. Gong , L. Li , H. Zhou , and Y. W. Mai , “Three‐Dimensional Nano‐Structural Design for Enhanced Damping Performance of Carbon fiber Reinforced Polymer Composites with Balanced Mechanical Performance,” Composites Part B: Engineering 298 (2025): 112351, 10.1016/j.compositesb.2025.112351.

[47] S. Liu , J. Wang , B. Zhang , et al., “Transformation of Traditional Carbon Fibers from Microwaves Reflection to Efficient Absorption via Carbon fiber Microstructure Modulation,” Carbon 219 (2024): 118802, 10.1016/j.carbon.2024.118802.

[48] H. Huang , G. Xue , J. Zhan , et al., “Polymer‐Based Flexible Wireless Sensors for Health Monitoring,” Nano‐Micro Letters 18, no. 1 (2026): 398, 10.1007/s40820-026-02233-5. 42252376 PMC13243171

[49] D. Liu , S. Liu , C. Chen , K. Wu , J. Ran , and Y. Zhang , “The Interface Anchoring Effect of Nickel Nanoparticles on Carbon Nanotube/Nickel/Copper Composite Wires with Ultra‐High Conductivity and Current‐Carrying Capacity,” Carbon 244 (2025): 120611, 10.1016/j.carbon.2025.120611.

[50] A. Darvishzadeh and K. Nasouri , “Manufacturing, Modeling, and Optimization of Nickel‐Coated Carbon Fabric for Highly Efficient EMI Shielding,” Surface and Coatings Technology 409 (2021): 126957, 10.1016/j.surfcoat.2021.126957.

[51] H. Guan and D. D. L. Chung , “Effect of the Planar Coil and Linear Arrangements of Continuous Carbon fiber Tow on the Electromagnetic Interference Shielding Effectiveness, with Comparison of Carbon Fibers with and without Nickel Coating,” Carbon 152 (2019): 898–908, 10.1016/j.carbon.2019.06.085.

[52] S. Ziaee , M. Montazer , and R. Bagherzadeh , “Flexible Copper/Nickel Carbon Composite Fabrics as Electrodes for Wearable PVDF/BaTiO_3_ Composite Piezoelectric Nanogenerators,” Composite Structures 357 (2025): 118868, 10.1016/j.compstruct.2025.118868.

[53] M. Yin , J. Luo , C. Yang , et al., “Stabilized Interface Design of Nickel‐Plated Carbon fiber Felts/Epoxy Composites with Co‐MOF toward Simultaneously Boosting Electromagnetic Interference Shielding and Mechanical Properties,” Composites Communications 54 (2025): 102260, 10.1016/j.coco.2025.102260.

[54] M. S. Kim , K. H. Kim , J.‐H. Kang , B.‐K. Choi , J. H. Park , and S. Y. Kim , “Highly Efficient EMI Shielding and Joule Heating at Low Filler Loadings via Optimization of Dispersion and Length Retention of Nickel‐Plated Carbon fiber Composites,” Composites Part A: Applied Science and Manufacturing 210 (2026): 110068, 10.1016/j.compositesa.2026.110068.

[55] W. Jang , D. Jang , J. Bang , et al., “Multi‐Layer Cement Composites for Absorption‐Dominant Electromagnetic Interference Shielding with Recycled Carbon Fibers,” Journal of Building Engineering 115 (2025): 114530, 10.1016/j.jobe.2025.114530.

[56] W. Zhou , Y. Li , J. Xu , Y. Ao , and M. Li , “Three‐Dimensional Interfacial Engineering of Carbon Fibers for Enhanced Mechanical Properties and Electromagnetic Shielding of Carbon Fiber Composites,” Polymer Composites 47, no. 10 (2026): 9002–9013, 10.1002/pc.70736.

[57] H. Moradi , K. Nasouri , and G. Askari , “Designing, Modeling, and Multi‐Response Optimization of Ni/Co‐Coated Carbon Fibers for Electromagnetic Shielding and Electrothermal Applications,” Journal of the Taiwan Institute of Chemical Engineers 172 (2025): 106116, 10.1016/j.jtice.2025.106116.

[58] Y. Wang , J. Wang , Z. Liu , S. Zheng , W. Lu , and Z. He , “Nanocone‐Shaped Ni‐Electroplated Carbon fiber Composite Films for Electromagnetic Shielding Applications,” Journal of Materials Research and Technology 27 (2023): 4625–4632, 10.1016/j.jmrt.2023.10.216.

[59] N. Jeong and D. Cho , “Effect of fiber Side‐Feeding on Various Properties of Nickel‐Coated Carbon‐Fiber‐Reinforced Polyamide 6 Composites Prepared by a Twin‐Screw Extrusion Process,” Journal of Composites Science 7, no. 2 (2023): 68, 10.3390/jcs7020068.

[60] K. Joshi , M. I. Arefev , and L. V. Zhigilei , “Generation and Characterization of Carbon fiber Microstructures by Atomistic Simulations,” Carbon 152 (2019): 396–408, 10.1016/j.carbon.2019.06.014.

[61] L. Martínez , R. Andrade , E. G. Birgin , and J. M. Martínez , “PACKMOL: a Package for Building Initial Configurations for Molecular Dynamics Simulations,” Journal of Computational Chemistry 30, no. 13 (2009): 2157–2164, 10.1002/jcc.21224. 19229944

