# Supplementary material for: Surface Neuronal‐Like Structuring of High‐Strength CFRP for Enhanced Electromagnetic Interference Shielding
Source: Adv Sci (Weinh). 2026 Aug 20;13(62):e77333. doi: 10.1002/advs.77333 (PMC13491364; doi:10.1002/advs.77333)
Supplement: Supplementary file 1 [file ADVS-13-e77333-s001.docx]

Supporting Information

Surface Neuronal-Like Structuring of High-Strength CFRP for Enhanced Electromagnetic Interference Shielding

Wei Cheng, Ben Jia, Shuhan Xiang, Biao Chen, Yongjun Zhang, Zhuo Liu*, Xiaopeng Wan, Heyuan Huang*

**Figure S1**. Ni grain size statistics for the sample electrodeposited at a current density of 0.04 A/cm²: (a) 10 min, (b) 20 min, (c) 25 min, (d) 30 min.

**Figure S2.** SEM images of coating thickness for samples electrodeposited at a current density of 0.04 A/cm² for 10 min (a, f), 20 min (b, g), 25 min (c, h), and 30 min (d, i), and at 0.02 A/cm² for 30 min (e, j).

**Figure S3**. (a) Variation curve of coating thickness with electrodeposition time under high current density; (b–e) EDS maps and corresponding elemental contents of the sample electrodeposited at 0.02 A/cm² without heat treatment; (f–i) EDS maps and corresponding elemental contents of the sample electrodeposited at 0.02 A/cm² combined with heat treatment at 700 °C.

**Figure S4**. Grain size statistics for samples prepared at a current density of 0.02 A/cm² under different heat treatment conditions: (a) 500 °C, (b) 600 °C, (c) 700 °C, (d) 700 °C with linear heating, (e) 800 °C, and (f) 900 °C. Except for (d), all samples were subjected to instantaneous heating.

**Figure S5.** Grain size statistics for samples subjected to furnace ramping to 700 °C: electrodeposited at 0.04 A/cm² for (a) 10 min, (b) 20 min, and (c) 30 min; and electrodeposited at 0.02 A/cm² for (d) 30 min.

**Figure S6.** Grain size diagrams of the surface Ni coating for samples electrodeposited at a current density of 0.04 A/cm² under different electrodeposition times combined with different transient heating conditions.

**Figure S7**. High-resolution XPS Ni 2p spectra with peak fitting for samples prepared at a current density of 0.02 A/cm² after instantaneous heat treatment at 500–900 °C (a–e) and after linear-ramp heat treatment at 700 °C (f), and those prepared at 0.04 A/cm² after instantaneous heat treatment at 500–900 °C (g–k) and after linear-ramp heat treatment at 700 °C (l).

**Figure S8**. High-resolution XPS O 1s spectra with peak fitting for samples prepared at a current density of 0.02 A/cm² after instantaneous heat treatment at 500–900 °C (a–e) and after linear-ramp heat treatment at 700 °C (f), and those prepared at 0.04 A/cm² after instantaneous heat treatment at 500–900 °C (g–k) and after linear-ramp heat treatment at 700 °C (l).

**Figure S9**. Schematic illustration of the sample preparation process and the MD modeling workflow.

**Figure** **S10**. Cross-sectional TEM morphologies and EDS elemental mappings of the unheat-treated carbon fiber sample electrodeposited for 30 min at a low current density of 0.02 A/cm²: (a) Cross-sectional bright-field (BF) TEM micrograph; (b) Overlaid EDS elemental mapping of four elements (C, O, Ni and W); (c) Elemental mapping of C; (d) Elemental mapping of Ni; (e) Elemental mapping of O; (f) Elemental mapping of W (protective layer).

**Figure S11.** High-resolution TEM-EDS characterizations of interfacial regions with different oxygen contents in the unheat-treated carbon fiber sample electrodeposited for 30 min at a low current density of 0.02 A/cm². (a–f) High-magnification bright-field (BF) micrograph of the oxygen-rich interfacial region and the corresponding elemental mappings of C, Ni, O and W (protective layer); (g–l) High-magnification bright-field (BF) micrograph of the oxygen-lean interfacial region and the corresponding elemental mappings of C, Ni, O and W (protective layer).

**Figure S12.** Cross-sectional TEM morphologies and EDS elemental mappings of the carbon fiber sample electrodeposited for 30 min at a low current density of 0.02 A/cm² followed by vacuum heat treatment with linear temperature ramping up to 700 °C: (a) Cross-sectional high-angle annular dark-field (HAADF) micrograph; (b) Overlaid EDS elemental mapping of four elements (C, O, Ni and W); (c) Elemental mapping of C; (d) Elemental mapping of Ni; (e) Elemental mapping of O; (f) Elemental mapping of W (protective layer).

**Figure S13.** High-resolution TEM-EDS characterizations of interfacial regions with different oxygen contents in the carbon fiber sample electrodeposited for 30 min at a low current density of 0.02 A/cm² followed by vacuum heat treatment with linear temperature ramping up to 700 °C: (a–f) High-magnification bright-field (BF) micrograph of the interfacial region with trace residual oxygen and the corresponding elemental mappings of C, Ni, O and W (protective layer); (g–l) High-magnification bright-field (BF) micrograph of the oxygen-free interfacial region and the corresponding elemental mappings of C, Ni, O and W (protective layer).
